# Identification of differentially expressed proteins of *Arthrospira* (*Spirulina) plantensis*-YZ under salt-stress conditions by proteomics and qRT-PCR analysis

**DOI:** 10.1186/1477-5956-11-6

**Published:** 2013-01-30

**Authors:** Huili Wang, Yanmei Yang, Wei Chen, Li Ding, Peizhen Li, Xiaokai Zhao, Xuedong Wang, Aiying Li, Qiyu Bao

**Affiliations:** 1 Institute of Biomedical Informatics/Zhejiang Provincial Key Laboratory of Medical Genetics, School of Life Sciences, Wenzhou Medical College, Wenzhou, 325035, China; 2 School of Environmental Science and Public Health, Wenzhou Medical College, Wenzhou, 325035, China; 3 College of Life Sciences, Central China Normal University, Wuhan, 450002, China

**Keywords:** *Spirulina plantensis*, *Arthrospira*, Metabolism pathway, Up-regulation, Salt-stress

## Abstract

*Arthrospira (Spirulina) platensis* as a representative species of cyanobacteria has been recognized and used worldwide as a source of protein in the food, which possesses some unusual and valuable physiological characteristics, such as alkali and salt tolerance. Based on complete genome sequencing of *Arthrospira (Spirulina) plantensis*-YZ, we compared the protein expression profiles of this organism under different salt-stress conditions (i.e. 0.02 M, 0.5 M and 1.0 M NaCl, respectively), using 2-D electrophoresis and peptide mass fingerprinting, and retrieved 141 proteins showing significantly differential expression in response to salt-stress. Of the 141 proteins, 114 *Arthrospira (Spirulina) plantensis*-YZ proteins were found with significant homology to those found in *Arthrospira* (76 proteins in *Arthrospira platensis* str. Paraca and 38 in *Arthrospira maxima* CS-328). The remaining 27 proteins belong to other bacteria. Subsequently, we determined the transcriptional level of 29 genes *in vivo* in response to NaCl treatments and verified them by qRT-PCR. We found that 12 genes keep consistency at both transcription and protein levels, and transcription of all of them but one were up-regulated. We classified the 141 differentially expressed proteins into 18 types of function categories using COG database, and linked them to their respective KEGG metabolism pathways. These proteins are involved in 31 metabolism pathways, such as photosynthesis, glucose metabolism, cysteine and methionine metabolism, lysine synthesis, fatty acid metabolism, glutathione metabolism. Additionally, the SRPs, heat shock protein and ABC transporter proteins were identified, which probably render *Arthrospira (Spirulina) plantensis*’s resistance against high salt stress.

## Introduction

Cyanobacteria is a phylum of bacteria which obtains energy through photosynthesis, which can be classified into several subgroups by morphology (unicellular or filamentous) or function (N2-fixing and non-N2-fixing) [1]. *Arthrospira (Spirulina) platensis* (ASP) is one of representative species of cyanobacteria, and has been harvested or cultured as a source of protein in the food [2-4]. Scientists found that this organism possesses some unusual and valuable physiological characteristics, such as alkali- and salt-tolerance. Especially, it can grow at salt concentrations of ~1.5-fold higher than in sea water [5,6], making it often dominates in lakes with high alkalinity.

At present, salt stress is becoming a serious environmental issue because 7% of the land, 20% of the arable land and 50% of irrigated land in the world are salinized. The agricultural production was reported to be dramatically reduced in approximate 33% by soil salinization [7]. Therefore, mechanism elucidation of salt-tolerance in plants would allow us to grow salt-tolerant plants using biological engineering techniques.

With the rapid development in molecular biology techniques, the salt-tolerance mechanism of cyanobacteria has been gradually clarified. The optimal Na^+^ concentration is 150–200 mM for the growth of cyanobacteria, and the cyanobacterium *Aphanothece halophytica* can grow well at a concentration of as high as 1000 mM NaCl [8]. It has been demonstrated that the sustained tolerance to high salt concentrations resulted from Na^+^/H^+^ reverse collaborative transportation system, hydrated ion channels, synthesis of some lipid membrane composition and salt-stress induced proteins [9,10].

For example, high salt-stress influences photosynthesis process of *Synechococcus* by inhibiting the expression of *psbA* gene encoded D1 protein, which is involved in the repair of PSII system [11]. Kanesaki and coworkers found that 28 genes specifically express in response to high salt-stress, 11 genes to high osmotic pressure, and 34 genes to both conditions in *Synechocystis sp*. PCC 6803 using the gene micro-array technology [12,13]. The expressed proteins in response to high osmotic pressure are mainly the metabolism-related lipoprotein A enzymes. However, they belong to hot shock proteins and glucosylglycerol-phosphate synthases. Moreover, some unknown proteins have been found to be highly expressed under adverse circumstance stress [14,15].

In the recent years, transcriptomics, proteomics and metabolomics technologies have been developed rapidly on the basis of cyanobacteria genomics studies under environmental stress conditions [16]. However, as for transcriptomic and proteomic analyses, the more advanced biological information technology is required to acquire the more reliable data [17]. The first commercial instrument for quantitative PCR (qPCR) created by American ABI Co., Ltd. in 1996 was thought to realize the detecting leap from traditional qualitative PCR to quantitative analysis. This technique can monitor slight changes of the whole biological system, and precisely quantify the gene expression level in the form of absolute or relative values. Consequently, high sensitivity, high accuracy, wide detection range and good repeatability led to wide application of quantitative PCR in quantitative gene expression study [18,19].

The present paper aims to analyze the protein expression of ASP-YZ using 2-dimensional gel electrophoresis (2-DE) and MALDI-TOF/MS to reveal the responses of ASP-YZ to salt-stress at molecular level. This study would allow us understanding the environmental adaptability of cyanobacteria, and provide basic clues to further clarify the mechanism of adverse circumstance-resistance in ASP.

## Material and methods

### Culture conditions and sample preparation

ASP-YZ supplied by Chinese Academy of Agricultural Sciences (Beijing, China) was cultured in 0.02 M NaCl Zarrouk medium at 30°C, light intensity of 8 klx and 75% humidity [20]. At logarithmic growth period, ASP-YZ cells were transferred into fresh Zarrouk medium supplemented with NaCl at three different salt concentrations (0.02, 0.5 and 1.0 M representing control, medium and high salt concentrations, respectively) and cultivated for 7 d. ASP-YZ growth curves are shown in Additional file 1: Figure S1. The harvested cells were rinsed three times with phosphate-based buffer, and then centrifuged at 9,000 rpm for 5 min to remove old and dead cells which were deposited at the tube bottom. The suspended cells were rinsed five times with ultra-pure water to remove NaCl completely. The cells for protein extraction were stored at −80°C for further analysis.

### Protein extraction

The harvested cells prepared as above were put in liquid nitrogen for 10–15 min, followed by grinding to fine powder. Soluble proteins were extracted by adding 1.5 mL of extraction buffer (10% trichloroacetic acid and 0.1% dithiothreitol (DTT) in acetone) to 100 mg lyophilized fine powder, followed by 1 min of Vortex agitation. The homogenate was kept at −20°C for 2–3 h, and then centrifuged at 35,000 g, 4°C for 15 min. The precipitates were resuspended with acetone containing 0.1% DTT and kept at −20°C overnight. After centrifugation, the precipitates were resuspended with acetone containing 0.1% DTT at −20°C for 1–2 h, then centrifuged at 35,000 g, 4°C for 15 min, and vacuum dried. After the resultant powder was resuspended in lysis buffer (8 M Urea, 4% NP-40, 0.8%Bio-lyte (pH 3.0-10.0), 40 mM Tris base (pH 8.5), 10 mM DTT, 1 mM PMSF (phenylmethyl sulfonylfluoride), 2 mM EDTA-Na_2_, the suspension was ultrasonically treated for 12 min in ice. The insoluble precipitates were removed by centrifugation at 35,000 g for 30 min at 4°C, and the protein concentration of the final supernatant was measured according to Bradford protein assay [21].

### Two-dimensional gel electrophoresis

For one-DE, the protein extract (22 μL) was diluted to a final concentration of 1000 μg/ml with an IEF rehydration solution, consisting of 17.5 μL DTT, 1.75 μL IPG (immobilized pH gradient buffer solution) and 309 μL R-Buffer (2 M sulfourea, 7 M urea, 4% CHAPS, 40 mM Tris, 2 mM TBP reductant and 0.2% Biolyte). Then, the above diluted protein extract (350 μL) was subjected to Immobiline DryStrips (pH, 4–7; Length, 17 cm; Amersham Bioscience), as shown in Additional file 1: Figure S2. Each experiment group contained three biological replicates, generating 9 individual samples. For the first-dimensional IEF (isoelectric focusing) based on pI value of each protein, the procedures were as follows: 50 V for 4–6 h, 500 V for 1 h, 1,000 V for 1 h, 8,000 V for 1 h, 8,000 V for 80,000 Vh and finally 500 V for 20 h. After this, strips were firstly equilibrated for 12 min in reducing solution (6 M urea, 50 mM Tris–HCl pH 6.8, 30% v/v glycerol, 2% w/v SDS and 2.5% w/v DTT). The strips were subsequently placed for another 15 min in alkylating equilibration buffer containing 2.5% (w/v) iodoacetamide instead of 2% iodoacetamide. Second dimension SDS-PAGE was run on homogeneous 12% T, 2.6% C (piperazine diacrylamide) polyacrylamide gels cast in glass plates. Electrophoresis was carried out at 20°C, and 1.0 W/Gel for 30 min and kept at 15 W/Gel until the dye front reached the bottom of the gel using Ettan-Dalt six unit. Gels were fixed overnight in 50% ethanol/5% acetic acid, and rinsed (5 gels/tray) four times (1 h each time) in 1,800 mL of deionized water. Following the washing, the gels were equilibrated in 1.9 g/L AgNO_3_ (1 L/tray) for 60 to 90 min. An additional 1-h wash in Na_2_CO_3_ (7.5 g/L) following the reducing step [22]. The protein spots were scanned by UNAX Powerlook 2100XL (Bio-Rad) and analyzed by ImageMaster 2D platinum 5.0 (Amersham Bioscience) according to manufacturer’s instructions.

### Image acquisition and data analysis

Images were properly cropped and optimized before performing the inter-gel matching of the standard protein maps. Before spot matching, the internal standard image gel with greatest number of spots was used as a master gel. The spot detection parameters were optimized by checking different protein spots in certain regions of the gel, which were then automatically detected, followed by visual inspection for removal or addition of undetected spots. Spot detection was refined by a manual spot edition where needed. The spots that were present on at least two gels of one treatment or control based on the image analysis were identified as expressed protein spots. The abundance of each protein spot was estimated by the percentage volume (Vol%), that is, the spot volumes were normalized as a percentage of the total volume in all the spots present in the gel to correct the variability because of loading, gel staining and destaining [23,24]. The percentage volumes were used to designate the significant differentially expressed spots (at least three-fold increase/decrease and statistically significant as calculated by Student’s *t*-test, at *P*<0.05). Triplicate gels were used for each sample. Only those with reproducible and significant changes were considered to be differentially expressed protein spots.

### Mass spectrometry analysis and database search

Differentially expressed protein spots were manually selected and excised, then de-stained in 50% ACN in 25 mmol/L NH_4_HCO_3_ at 37°C for 30 min. Next, 50 μL ultra-pure H_2_O and 50 μL 50% ACN were added, followed by 100 μL of 100% ACN. The gels were rehydrated in a 5 μL of a trypsin (Promega, Madison, USA) solution (20 μg/ml in 25 mmol/L NH_4_HCO_3_) for 30 min. Next, 20 μL cover solution (25 mmol/L NH_4_HCO_3_) was added, and digestion took place overnight at 37°C. The supernatants were transferred into another tube, and the gels were extracted once with 50 μL extraction buffer (67% ACN and 5% TFA). The peptide extracts and the supernatant of the gel spot were combined and then completely dried. Samples were re-suspended in 5 μL 0.1% TFA followed by mixing in 1:1 ratio with a matrix consisting of a saturated solution of CHCA in 50% ACN containing 0.1% TFA. The 1:1 mixture was spotted on a stainless steel sample target plate. The peptide samples were analyzed with a MALDI-TOF/TOF Proteomics Analyzer (Bruker Daltonics, Bremen, Germany). A combined search (MS plus MS/MS) was performed using Bruker Daltonics BioTools 3.0 software (Bruker Daltonics Inc). The TOF spectra were recorded in positive ion reflector mode with a mass range from 800 to 4000 Da. About eight subspectra with 60 shots per subspectrum were accumulated to generate one main TOF spectrum.

Data were searched on the Internet using a Mascot search engine (Matrix Science Ltd., London, UK) against all entries in the NCBInr database, which contained accessible public protein sequences (http://www.ncbi.nlm.nih.gov/nuccore/NZ_ACSK01001820.1/*Arthrospira platensis* str. Paraca NZ_ACSK01001820) and (http://www.ncbi.nlm.nih.gov/nuccore/NZ_ABYK00000000.1/*Arthrospira maxima* CS-328), and the unpublished *Arthrospira platensis* database. The unpublished database was built by our group after nearly complete sequencing and annotation of ASP-YZ. All peptide masses were assumed monoisotopic and [M+H]^+^. The other parameters used for search were as follows: taxonomy, other bacteria; enzyme, trypsin; the fixed modification; carbamidomethyl (C); the variable modification, Glu->pyro-Glu (N-term Q) and oxidation (M); mass toll=±100 pm. The confidence in the peptide mass fingerprinting matches (*P* < 0.05) was based on the MOWSE Score and confirmed by the accurate overlapping of the matched peptides with the major peaks of the mass spectrum. Only the significant hits, as defined by a MASCOT probability analysis (*P* < 0.05), were accepted. These differential proteins were grouped in their respective metabolic pathways using COG software online (ftp://ftp.ncbi.nih.gov/pub/COG, E-value < 0.0001).

### qRT-PCR analysis of the differentially expressed genes

The primer sets, shown in Additional file 1: Table S1, were designed for amplification of 16S rRNA and differentially expressed genes using Premier 5.0 software, and synthesized by Shanghai Sonny Biological Technology Co., Ltd. (Shanghai, China).

To validate the differential proteins of ASP-YZ at transcriptional level *in vivo*, qRT-PCR was performed using the StepOne™ RT-PCR System (Applied Biosystem, USA). Total RNAs were extracted from ASP-YZ cultured in the control and medium salt concentrations using RNeasy Plant Mini Kit (Qiagen, Germany) according to manufacturer instructions and treated with Dnase I by RNase-Free Dnase Set (Qiagen, Germany). cDNA was obtained by reverse transcription using PrimeScript RT-PCR Kit (TaKaRa, Japan), following the procedures composed of an initial denaturation time of 5 min at 95°C, 35 cycles of amplification comprising of a denaturation step for 1 min at 95°C, and the annealing and extension temperatures as listed in Additional file 1: Table S2. Relative quantification of the targets in each sample was carried out using the signal of 16S rRNA as a control.

The copy numbers in two samples were normalized, and the differentially expressed levels were then calculated using the following equation:

$$\text{Folds}=\frac{G_{t}\times C_{h}}{C_{t}\times G_{h}}$$

where G_t_, G_h_, C_t_ and C_h_ represent target genes of the treatments, housekeeping genes of the treatments, target genes of the controls and housekeeping genes of the controls, respectively.

## Results

### 2-DE profiles of total ASP-YZ proteins in response to different salt concentrations

**Figure 1:**
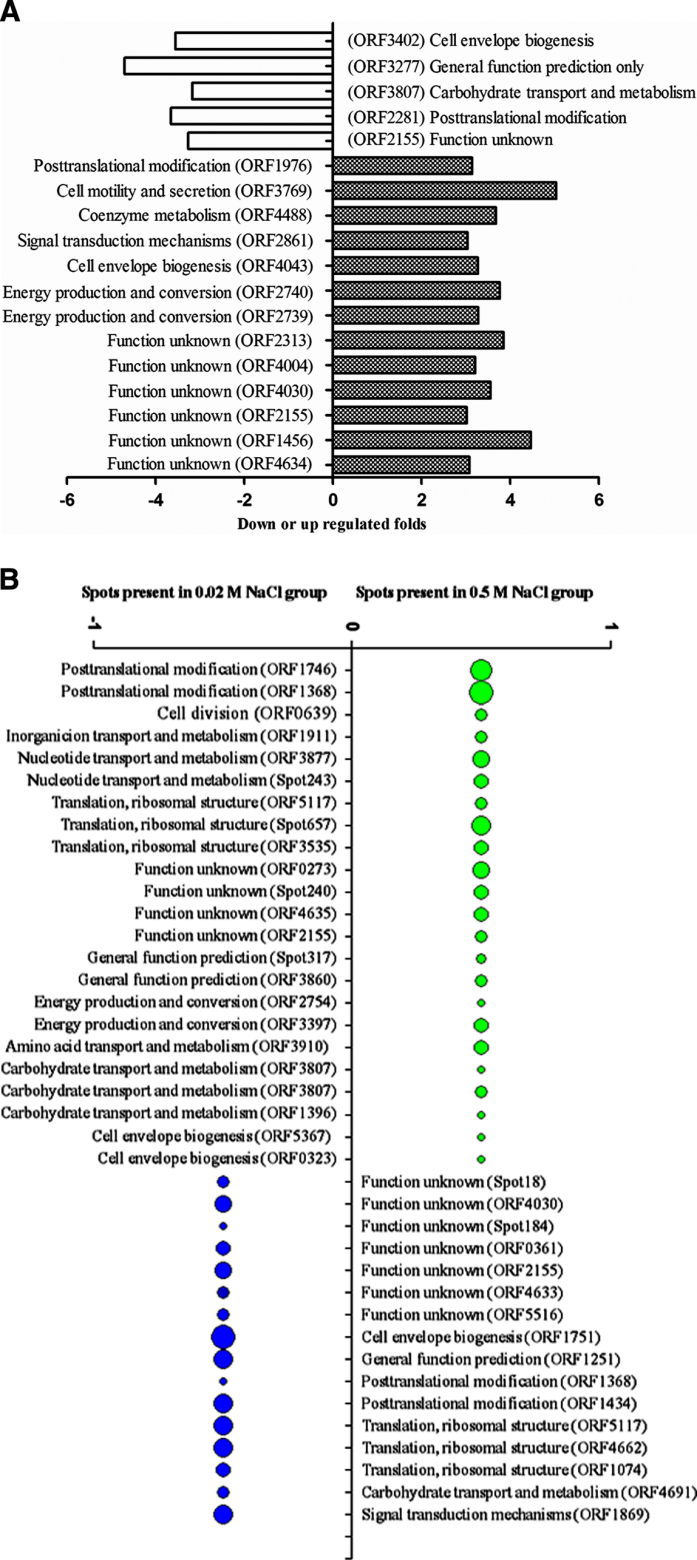
**The differential protein points between medium salt (0.5 M NaCl) treatment and the control (0.02 M NaCl).** (Note: **(A)** The differential protein points with >3.0-fold changes in medium salt (0.5 M NaCl) treatment compared with the control (0.02 M NaCl). **(B)** The protein-spots of absent or present difference between the two groups (the relative abundance (Vol%) of “present” protein calculated by the ratio of volume of each protein spot to volume of total protein spots in the whole gel,ranging from 0.928% to 8.06% as shown in Additional file 1: Table S3-B).

**Figure 2:**
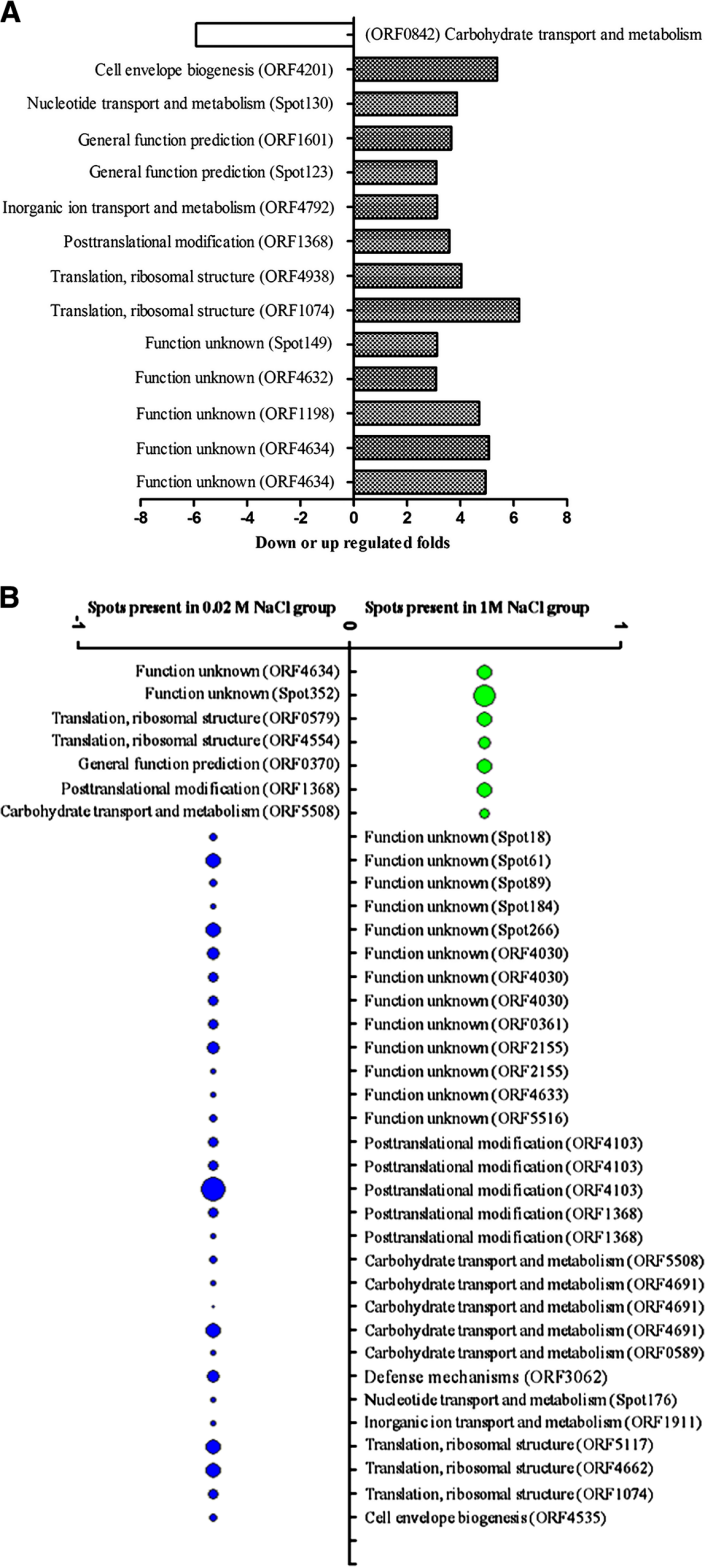
**The differential protein points between high salt (1.0 M NaCl) treatment and control (0.02 M NaCl).** (Note: **(A)** The differential protein points with >3.0-fold changes in high salt (1.0 M NaCl) treatment group compared to control (0.02 M NaCl). **(B)** The protein-spots of absent or present difference between the two groups (the relative abundance (Vol%) of “present” protein calculated by the ratio of volume of each protein spot to volume of total protein spots in the whole gel,ranging from 1.208% to 11.872%, as shown in Additional file 1: Table S3-D).

The expressed protein profiles of ASP-YZ under three salt concentrations of 0.02 M (control), 0.5 M and 1.0 M were analyzed by 2-DE (with triplicate gels each salt concentration, see Additional file 1: 3 S2), followed by the statistical evaluation of the protein spots using ImageMaster 2D platinum 5.0 software for isoelectric point, molecular weight, relative expression abundance and protein-spot matching information etc. The overall protein numbers of ASP-YZ cultured in different salt concentrations appeared similar. Identical protein spots in the paired gels between different salt concentrations were also analyzed using the lower salt concentrations gels as the reference in each pair. There were 583 protein spots matched between the control (0.02 M NaCl) and 0.5 M NaCl gels (with correlation coefficient of 0.937 and matching rate of 83.90%), 467 between the control and 1.0 M NaCl gels (with correlation coefficient of 0.948 and matching rate of 72.68%) and 532 between 0.5 M NaCl and 1.0 M NaCl gels (with correlation coefficient of 0.964 and matching rate of 78.69%). Eighteen protein spots and their fold changes were found at the medium salt concentration (0.5 M), which is over 3-fold higher differential expression compared to the control (0.02 M) (Figure 1A). In addition, 23 proteins were solely present under the medium salt treatment, while 16 proteins were solely present under the control (Figure 1B). There were 14 protein spots which are over 3-fold higher differential expression at the high NaCl concentration (1.0 M) compared to the control (Figure 2A). In addition, 7 proteins were solely present under the high salt treatment, while 30 proteins were solely present under the control (Figure 2B).

**Figure 3:**
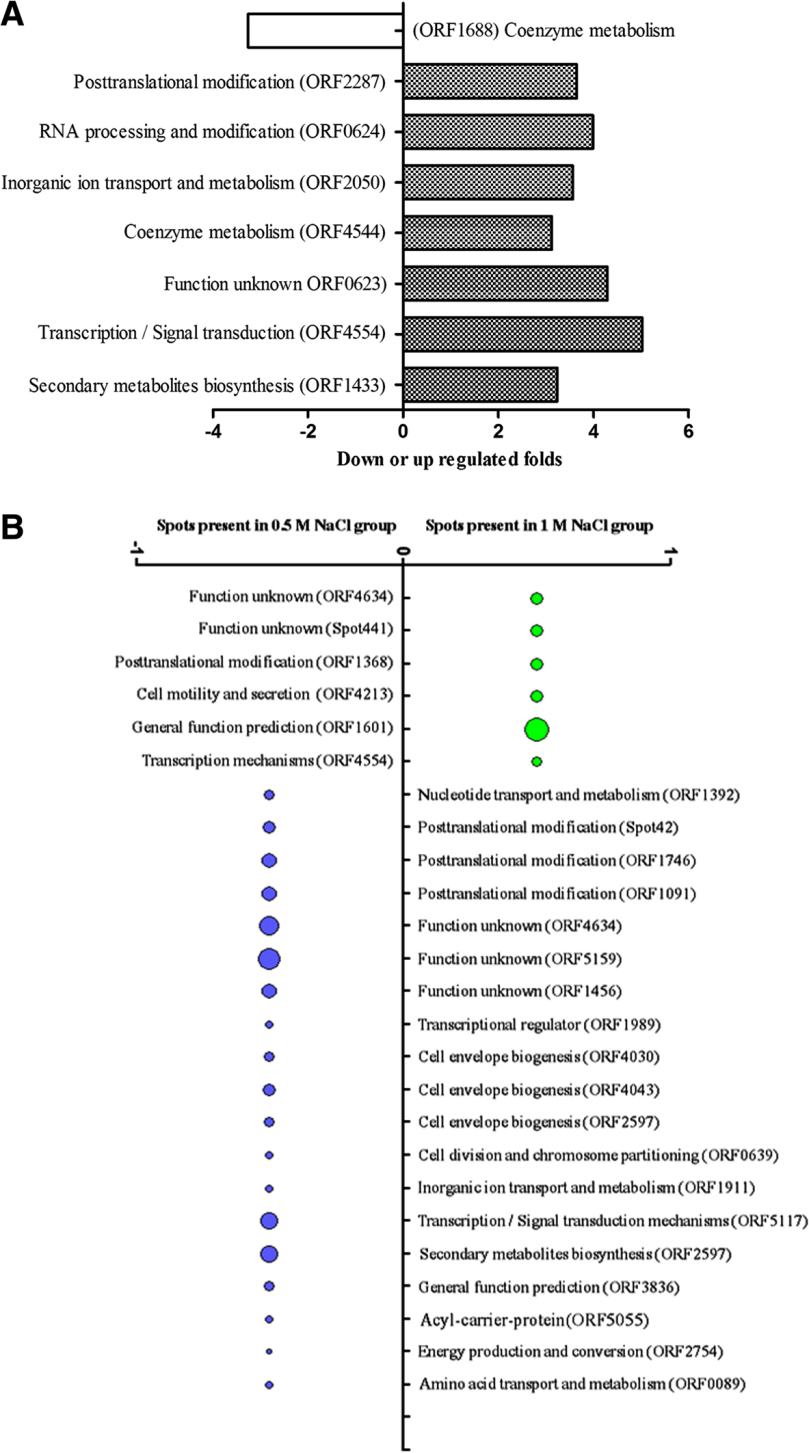
**The differential protein points between high salt (1.0 M NaCl) treatment and medium salt (0.5 M NaCl) treatment.** (Note: **(A)** The differential protein points with >3.0-fold changes in high salt (1.0 M NaCl) treatment compared to medium salt (0.5 M NaCl) treatment. **(B)** The protein-spots of absent or present difference between the two groups (the relative abundance (Vol%) of “present” protein calculated by the ratio of volume of each protein spot to volume of total protein spots in the whole gel, ranging from 2.170% to 22.854%, as shown in Additional file 1: Table S3-F).

By comparing the differential protein expression in the medium and high salt concentrations, 8 protein spots were found to express differentially by >3-fold higher at 0.5 M NaCl concentration than at 1.0 M concentration (Figure 3A). In addition, 6 protein spots were solely present under the medium salt treatment, while 19 protein spots were solely present under the high salt treatment (Figure 3B). In total, 141 differentially expressed protein spots were obtained from the control (0.02 M), medium (0.5 M) and high (1.0 M) salt-stress conditions (see Additional file 1: Table S3 A-F).

### PMF of the differential protein-spots and results of MALDI-TOF/MS analysis

In order to get more information on differential proteins, 141 differential spots were identified using MALDI-TOF/MS analysis. By MASCOT searching, these positive protein spots could be attributed to 82 classes, among which 76 proteins exhibit high homology with those in *Arthrospira platensis* strain Paraca, 38 proteins in *Arthrospira maxima* CS-328 and the remaining 27 proteins belong to bacteria.

**Figure 4:**
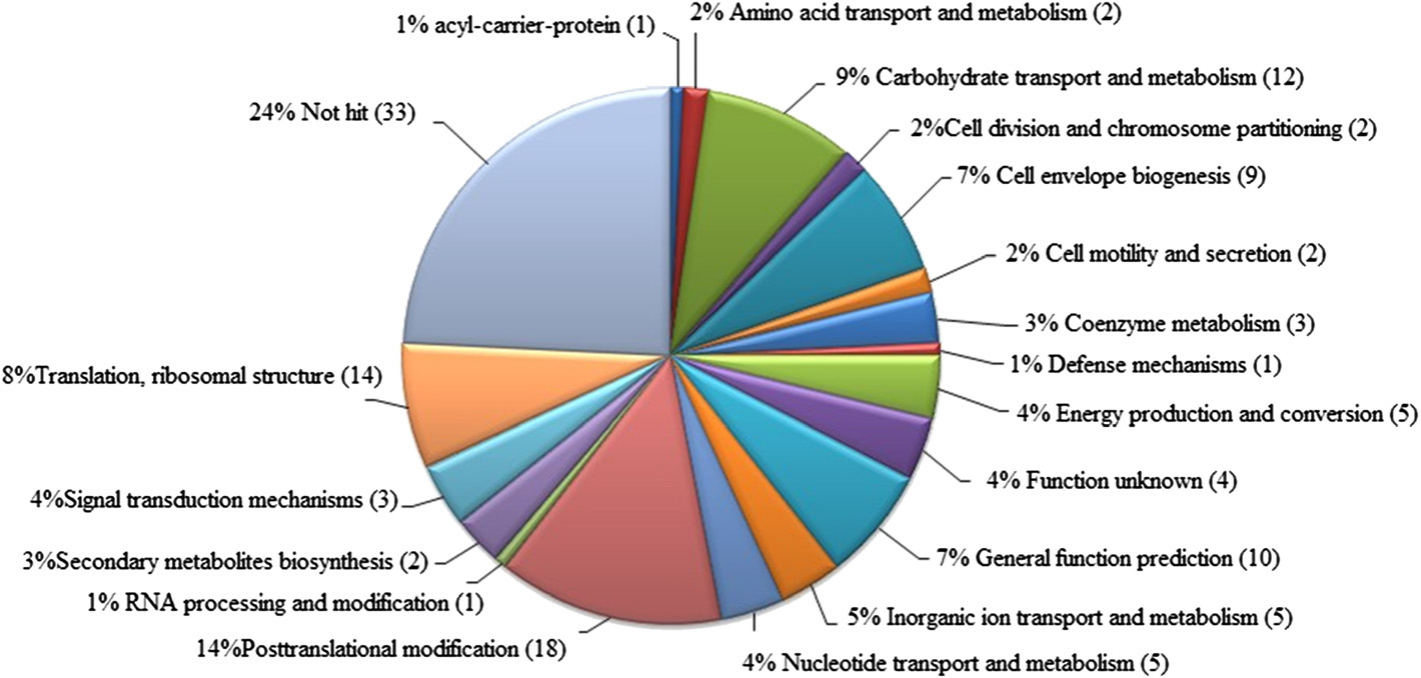
COG functional classification of 132 positive differential expression proteins.

By comparing the theoretical and actual values of PI and isoelectric point acquired by MS and Gel Map Analytical Software, we performed COG function prediction and classified these 132 positive proteins into 18 functional categories (Figure 4). The detailed information on 141 differentially expressed proteins is summarized in Additional file 1: Table S3 (A-F) and Table S4 (A-F).

### qRT-PCR analysis of the differentially expressed genes

#### Selection of differentially expressed genes

We selected the typical 29 genes for further validation, which are possible salt-tolerant genes predicted by previous studies [25-31], and the present results by metabolic pathway positioning through KEGG analysis, whose functions were involved in photosynthesis, glycolytic pathway, oxidative phosphorylation, amino acid and fatty acid synthetic metabolic pathway. Table 1 summarizes the information, annotation and metabolic pathway of 29 genes.

**Table 1:** Annotation and proposed metabolic pathways of the differential protein spots obtained from the MS-searching

**Spot**	**Spot**	**ORF**	**Accession no.**	**Gene product (best hit in NCBInr database)**	**MW (Da) (T/E)**	**PI (T/E)**	**Matched peptides**	**Cov**	**MASCT Score**	**E-value**	**Fold change**
**General function**											
CK701	M718	3277	ZP_03275759	FAD-dependent pyridine nucleotide disulphide oxidoreductase [*Arthrospira maxima* CS-328]	47147/42726	6.67/6.49	6	20%	78	0.018	−4.70
**Posttranslational modification, protein turnover, chaperones**											
CK275	M305	2281	ZP_06380867	Peptidyl-prolyl cis-trans isomerase, cyclophilintype[*Arthrospira* str. Paraca]	23921/28931	4.74/4.49	6	36%	75	0.036	−3.65
M908	H653	2287	ZP_03274434	Molecular chaperone DnaK [*Arthrospira maxima* CS-328]	62452/57079	4.70/5.57	27	38%	150	1.00E-09	3.65
**Fructose and mannose metabolism**											
CK461	H-N	4535	ZP_06381408	GDP-L-fucose synthase [*Arthrospira* str. Paraca]	35652/35258	5.59/6.14	23	53%	260	1.00E-20	/
**Fatty acid biosynthesis**											
CK361	H-N	5055	ZP_06383266	Enoyl-(acyl carrier protein) reductase [*Arthrospira* str. Paraca]	27751/31814	5.57/5.83	11	37%	79	0.013	/
M344	H224	1433	YP_002521548	3-oxoacyl-[acyl-carrier protein] reductase [*Thermomicrobium roseum* DSM 5159]	28631/31269	6.54/5.34	9	34%	73	0.049	3.24
**Photosynthesis - antenna proteins**											
CK47	H-N	4103	ZP_03271327	Phycobilisome core component [*Arthrospira maxima* CS-328]	17433/14807	6.26/5.74	14	84%	165	3.30E-11	/
CK342	M394	2155	ZP_06382427	Phycobilisome linker polypeptide [*Arthrospir*a str. Paraca]	29450/31199	9.25/5.68	15	55%	218	1.6E-16	−3.26
CK423	H-N	4633	ABV01983	Cpch [*Arthrospira* Sp-16]	30852/33929	7.82/6.29	25	60%	295	3.30E-24	/
CK467	H342	4632	ABB84420	Cpci [*Arthrospira* Sp-5]	32790/35621	8.33/4.59	19	53%	150	1.00E-09	3.09
CK-N	H69	4634	ZP_06380686	Phycocyanin, alpha subunit [*Arthrospira maxima* CS-328]	17703/19259	5.82/5.89	10	58%	91	0.00076	/
CK-N	M252	4635	ABD64607	Phycocyanin beta chain [*Arthrospira*]	18506/28381	5.19/4.58	11	55%	98	0.00016	/
**Purine metabolism**											
CK-N	M137	1911	ZP_06383116	Adenylylsulfate kinase [*Arthrospira* str. Paraca]	19897/22320	5.22/5.46	16	79%	200	1e-14	/
**Lysine biosynthesis**											
CK-N	M463	3910	ZP_06382240	Dihydrodipicolinate synthase [*Arthrospira* str. Paraca]	30869/34837	5.20/5.80	22	51%	141	8.3e-09	/
CK500	M548	4030	ZP_03276569	Diaminopimelate epimerase [*Arthrospira maxima* CS-328]	32452/37662	9.22/6.83	39	76%	310	1e-25	3.56
**Glutathione metabolism**											
CK517	H384	4938	ZP_06381009	Glutathione synthetase [*Arthrospira* str. Paraca]	36159/38491	5.93/6.64	15	43%	100	0.0001	4.04
**Glycolysis/Gluconeogenesis**											
CK-N	M595	3807	ZP_03274253	Glyceraldehyde-3-phosphate dehydrogenase, type I [*Arthrospira maxima* CS-328]	36534/39635	6.07/5.26	24	61%	110	0.00001	/
**Cysteine and methionine metabolism**											
M778	H545	1688	ZP_06381331	Adenosylhomocysteinase [*Arthrospira* str. Paraca]	46667/45550	5.64/5.98	25	52%	245	3.3e-19	3.26
**Oxidative phosphorylation**											
CK145	M140	2740	ZP_06381970	F-type H^+^-transporting ATPase subunit delta [*Arthrospira* str. Paraca]	20108/22132	6.15/6.79	15	53%	116	2.6e-06	3.77
CK109	M93	2739	ZP_03274295	F-type H^+^-transporting ATPase subunit β[*Arthrospira maxima* CS-328]	19538/17970	5.14/5.04	13	59%	114	4.2e-06	3.28
**ABC transporters**											
M800	H564	2050	ZP_03275033	ABC-type nitrate/nitrite transport system substrate-binding protein [*Arthrospira maxima* CS-328]	48859/47107	4.68/4.46	26	50%	235	3.30E-18	3.57
**Protein export**											
M-N	H185	4213	YP_798213	Signal recognition particle subunit SRP54[*Leptospira borgpetersenii serovarHardjobovis* L550]	49178/28565	7.08/5.46	8	24%	74	0.046	/
**Transcriptional regulator**											
M111	H-N	1989		Transcriptional regulator, abrb family [*Arthrospira maxima* CS-328]	15532/19479	8.49/4.22	11	44%	90	0.0011	/
**Function unknown**											
CK934	M-N	1251	ZP_06381540	Hypothetical protein aplap_07632 [*Arthrospira* str. Paraca]	67407/65336	5.66/6.20	19	42%	166	2.6E-11	/
CK424	M-N	5516	ZP_03272395	Conserved hypothetical protein [*Arthrospira maxima* CS-328]	41116/34016	6.04/6.08	13	50%	205	3.3E-15	/
CK283	M-N	3023	ZP_06383801	Hypothetical protein AplaP_19221 [*Arthrospira platensis* str. Paraca]	23973/29221	7.03/6.22	9	58%	94	0.00044	/
CK202	H127	1198	ZP_06382670	Hypothetical protein aplap_13413 [*Arthrospira* str. Paraca]	29241/25864	8.15/5.52	11	54%	103	5.2e-05	4.70
CK-N	M175	273	ZP_06384609	Hypothetical protein aplap_23383 [*Arthrospira* str. Paraca]	20229/25353	5.93/5.71	9	49%	121	8.3e-07	/
M839	H591	624	ZP_06384828	Hypothetical protein aplap_24525 [*Arthrospira* str. Paraca]	37661/50381	4.91/5.25	27	61%	226	2.60E-17	3.99

Note: (1) 0.02 M NaCl for control group (CK); 0.5 M NaCl for medium salt treatment (M); and 1.0 M NaCl for high salt treatment (H).

(2) CK-N, M-N and H-N represent no protein-spots are observed in control, medium and high salt treatment groups, respectively.

(3) The grey mark indicates up-regulation of proteins.

(4) The negative values in fold column indicate the down-regulation.

(5) T/E indicates theoretical values/experimental values.

#### The purity and concentration of total RNA

Through agarose gel electrophoresis, the extracted RNA in both control and medium salt treatment showed a good integrity and a high purity, and had no degradation (see Additional file 1: Figure S3). The OD_260_/OD_280_ values were 2.09 and 2.03, and the RNA concentrations were 441.2 and 113.6 ng/μL, respectively, in the control (0.02 M) and medium salt (0.5 M) treatment.

#### Standard curve of qRT-PCR

For the 29 salt-tolerant genes, the qRT-PCR technique was used to further confirm consistency between their transcriptional level and gene expression. All of the melting curves showed a single peak, suggesting a good specificity (see Additional file 1: Figure S4). The non-detectable fluorescence signal in the negative control indicates that the reaction system was not polluted during the analysis. As shown in Additional file 1: Table S5 and Table S6, the calibration curves, regression coefficients and amplification efficiencies of 16S rRNA (control) and target genes indicate efficient amplification with high specificity.

#### The relative transcription level of the genes using qRT-PCR

**Figure 5:**
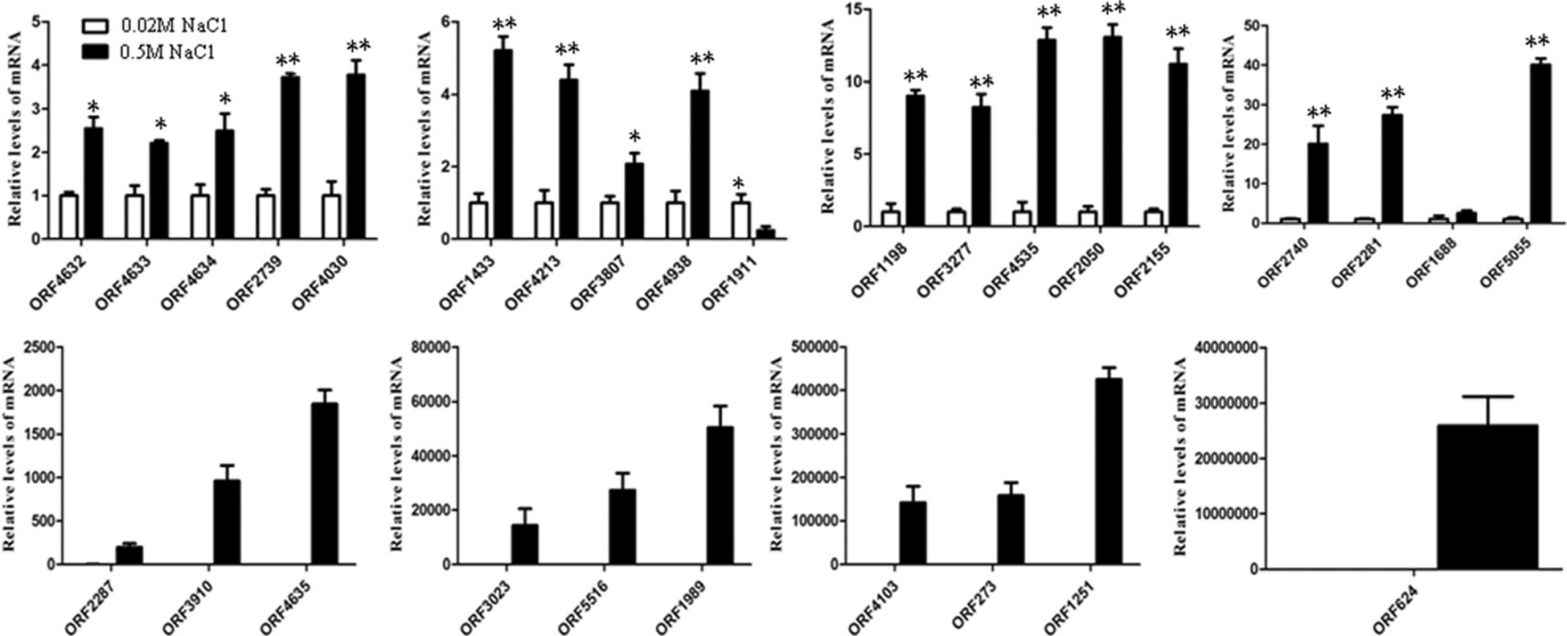
**qRT-PCR identification of 29 differential expression genes between the control (0.02 M NaCl) and the medium salt (0.5 M NaCl) treatment.** (Note: * indicates the significant difference, at p<0.05 level, of the transcriptional levels between medium salt treatment (0.5 M NaCl) and the control (0.02 M NaCl), and ** indicates the extremely significant difference at p<0.01 level).

The relative mRNA transcription level of the positive proteins detected by qRT-PCR for the control and medium salt group is shown in Figure 5. Under salt-stress conditions, the transcription of 28 genes was up-regulated, while only one gene transcription level was down-regulated. Of the 28 up-regulated genes, the genes with 2 to 3-fold transcription level were ORF4632, ORF4633, ORF4634, ORF3807 and ORF1688. Additionally, the genes up-regulated the transcription level to a higher extent included ORF2739 and ORF4030 (>3-fold), ORF4138 and ORF4213 (>4-fold), ORF1433, ORF1198 and ORF3277 (5–10 times), ORF4535, ORF2050, ORF2740, ORF2281, ORF5055 and ORF2155 (>10-fold), and ORF2287, ORF1989, ORF4103, ORF273, ORF5516, ORF1251, ORF3023, ORF624, ORF3910 and ORF4635 (>100-fold), respectively.

More interestingly, the fold change of ORF1989, ORF4103, ORF273, ORF5516, ORF1251, ORF3023, ORF624 and ORF4635 with up-regulated transcription level reached up to more than 10,000-fold. Of the 29 target genes, the transcription of only ORF1911 gene was down-regulated by ca. 5-fold.

Of the 29 target genes, the protein expression of 17 genes was consistent with gene transcription, representing 58.62% of the total target genes. The genes, with transcription levels of > 3-fold, were ORF4030, ORF3910, ORF4935, ORF2740, ORF2739, ORF1433, ORF2287, ORF2050, ORF4213, ORF4635, ORF273, ORF1189 and ORF624, respectively. The remaining 12 genes showed inconsistent protein expression with transcription level, which is 41.38% of the total target genes. The comparison of transcription and protein expression of the target genes is summarized in Table 2.

**Table 2:** The comparison between qPCR and proteomic analyzing result

**ORF**	**Fold change from qPCR results**	**Fold change from proteomic results**	**Consistency**
3277	UR >8	DR>4(0.5 M and control)	No
2281	UR >27	DR>3(0.5 M and control)	No
4535	UR >12	DR>3(1.0 M and control)	No
5055	UR >40	DR>3(0.5 M and 1.0 M treatment)	No
4103	UR >142017	DR>3(1.0 M and control)	No
1911	DR>4	DR>3(0.5 M and control)	No
4633	UR >2	DR>3(0.5 M and control)	No
2155	UR >11	DR>3 (0.5 M and control)	No
4030	UR >3	UR>3(0.5 M and control)	Yes
1989	UR >50406	DR<3(0.5 M and 1.0 M treatment)	No
1251	UR >425003	DR<3(0.5 M and control)	No
5516	UR >27288	DR<3(0.5 M and control)	No
3023	UR >14294	DR<3(0.5 M and control)	No
3910	UR >960	UR<3(0.5 M and control)	Yes
4938	UR >4	UR>4(1.0 M and control)	Yes
3807	UR >2	UR<3(0.5 M and control)	Yes
1688	UR >2	UR>3(0.5 M and 1.0 M treatment)	Yes
2740	UR >20	UR>3(0.5 M and control)	Yes
2739	UR >3	UR>3(0.5 M and control)	Yes
1433	UR >5	UR>3 (0.5 M and 1.0 M treatment)	Yes
2287	UR >194	UR>3(0.5 M and 1.0 M treatment)	Yes
2050	UR >13	UR>3(0.5 M and 1.0 M treatment)	Yes
4213	UR >4	UR>3(0.5 M and 1.0 M treatment)	Yes
4632	UR >2	UR>3(1.0 M and control)	Yes
4634	UR >2	UR>3(1.0 M and control)	Yes
4635	UR >1846	UR>3(0.5 M and control)	Yes
273	UR >158255	UR>3(0.5 M and control)	Yes
1198	UR >9	UR>4(1.0 M and control)	Yes
624	UR >25867164	UR>3(0.5 M and 1.0 M treatment)	Yes

UR and DR indicate up-regulation and down-regulation, respectively.

Finally, we performed the metabolic pathway positioning through KEGG analysis, and found 13 genes with >3-fold up-regulated protein under medium salt-stress (Figure 1A). The 13 genes were involved in photosynthesis, glycolytic pathway, oxidative phosphorylation, amino acid and fatty acid synthetic metabolic pathway. Additionally, ORF4213 and ORF2287 function in signal recognition particle (SRP) subunit and molecular chaperone DnaK (Hsp70). Moreover, the 7 genes with unknown function were found.

## Discussion

Cyanobacteria’s resistance to highly salty environments has been triggering great research interest to reveal the mechanism. Some findings suggested that osmotic stress as a main factor could result in decreasing of water content in cytoplasm and an increase in intracelluar salt concentration [32]. In the current study, we identified some up-regulated proteins in ASP-YZ at different salt concentrations by proteomics techniques, suggesting that up-regulation of the protein is a response to high salt environment. ASP-YZ acclimates to high salt environment probably through modulating osmotic regulation, because Glyceraldehyde-3-phosphate dehydrogenase (ORF3807, Table 1), the key enzyme of carbohydrate and amino acid metabolism, is detected. It has reported that generation of more trehalose is required to resist external osmotic pressure to protect the intracellular macromolecule stability [33]. We speculate that salt-stress might result in the signal recognition particle (SRP) to synthesize more glucose, fructose and betaine. The synthesis of these energy substances can maintain intracellular pressure by balancing the osmotic pressure caused by salt-stress.

Under salt-stress conditions, oxygen free radicals in ASP-YZ cells are expected to accumulate rapidly, and are harmful to cells if they are not removed timely [34]. We identified some up-regulated genes, at both protein and transcription levels, (Glutathione synthetase, ORF4938, Table 1) involved in glutathione synthetase in ASP-YZ, suggesting that salt-stress can enhance biosynthesis of intracellular glutathione, which in turn helps cells to clean oxygen free radicals, and to maintain the stability of intracellular environment [35].

The unsaturated fatty acid in thylakoid membrane of cyanobacteria has been found to play an important role in the process of adaptation to salt stress, by relieving the inhibition of synthesis and activity of Na^+^/H^+^ transporter caused by salt-stress [36]. The up-regulation of 3-oxoacyl-[acyl-carrier protein] reductase (ORF1433, Table 1), at both protein and transcription levels, might promote the photosynthesis under salt-stress conditions, which accelerates the active expulsion of Na^+^ through Na^+^/H^+^ transporter and thus decreases ion poisoning [37,38].

The transcription level of gene ORF4213 encoding signal recognition subunit SRP54 increased by >4-fold, which is in consistent with up-regulation of protein expression detected by 2-DE. The intracellular protein orientation transfer is an important link among protein quantity control, protein recognition and protein transport system, which is mediated by SRP [39]. SRPs are RNA-protein complexes, which can identify existence of the signal peptide in the nascent polypeptide chain and thus can mediate the recognition and transport of the secretory and membrane proteins [40]. Therefore, cell physiological metabolism and lesion are associated with protein targeting transport. It is predictable that expression of some SRP proteins (SRP54, ORF4213, Table 1) in response to salt-stress results from acceleration of the intracellular nascent peptide synthesis and enhancement of identification and transporter activity of the secretory and membrane proteins [41].

Under salt-stress conditions, many cyanobacteria can induce the expression of salt-stress proteins, in proportional increase to the tolerance to salt [42]. The expression of stress proteins and molecular chaperone Dnak in ASP-YZ were also found to be up-regulated in the present study, making major contributions to promote the correct folding of intracellular protein and the tolerance to salt.

Under salt-stress, the up-regulated enzymes in ASP-YZ include peptidyl-prolylcis-trans isomerase, cyclophilin type (encoded by ORF2281), diaminopimelate epimerase (encoded by ORF4030), enoyl-[acyl-carrier protein] reductase (ORF5055), transcriptional regulator, abrB family (ORF1989) and FAD-dependent pyridine nucleotide-disulphide oxidoreductase (ORF3277). Of the above 5 genes, up-regulation of 4 genes (ORF-2281, –5055, –1989 and −3277) was observed at transcription level, while down-regulation of them at protein level. Obviously, the salt-tolerant mechanism of ASP-YZ is not solely mediated through up or down-regulation of genes, at transcription or translation level, in one metabolite or one metabolic pathway. It should be a perplexing result of the cross-regulation of many physiological, biochemical and metabolic pathways.

It is worthy to note that each protein spot in gel does not necessarily accord to one kind of protein. The above phenomenon lies in: (1) the same gene has different expression products, (2) the same protein can form different spots due to different structural modification, and (3) the same protein can produce multiple protein fragments due to degradation. Overall, the above factors can lead to several protein spots attributed to the same protein (the same DNA Open Read Frame). Nevertheless, the protein spot changes in 2-DE images imply the change of protein (subunits), revealing that this kind of change (gene expression diversity and protein post-translational modification) is unique advantage of 2-DE and proteomic techniques.

Some ASP-YZ genes, showing inconsistency between transcription and translation level, do not account for their independence on salt tolerance, and perhaps their major roles are regulatory effects including transcriptional regulation, differential processing of RNA transcript and differential translation of mRNA [43,44]. Regulation of gene transcription and translation could be controlled by activation and transformation of gene structure, initiation of transcription, post-transcriptional processing and transport, mRNA degradation, translation and post-translational processing and protein degradation. The degradation of mRNA transcripts is an important reason to cause the inconsistency. Some inducible gene transcripts could be degraded immediately after translation and even in the course of translation [45]. The inconsistency between transcriptional and translational levels is influenced by many factors, and thus the further verification is required to elucidate its mechanisms.

Additionally, we detected that seven genes with unassigned functions was up-regulated, at transcription level, to a remarkably high extent, for instance, up-regulated by 9-fold for ORF1198, 1×10^4^-fold ORF3023, 2×10^4^-fold for ORF5516, 15×10^4^-fold for ORF273, 42×10^4^-fold for ORF1251, and 2586×10^4^ for ORF624 genes, respectively. It is of great importance to predict the role of these unknown candidate genes induced by salt-stress. The expression of some genes with unknown function was found to be down-regulated obviously, including ORF3277, ORF2281, ORF5055, ORF1251, ORF5516 and ORF3023. Therefore, it is necessary to study extensively the expression regulation mechanism of these genes at the transcriptional level under salt-stress conditions.

In a conclusion, we identified some interesting genes in response to salt-stress by proteomic analysis. To elucidate the salt-tolerant mechanism in ASP-YZ, the data in the present study will promote us to investigate further the expression regulation of these target genes.

## Abbreviations

ASP: *Arthrospira (Spirulina) platensis*; KEGG: Kyoto Encyclopedia of Genes and Genomes; COG: Cluster of Orthologous Groups of Proteins; SRP: Signal Recognition Particle; 2-DE: 2-Dimensional Gel Electrophoresis; PMSF: Phenylmethyl Sulfonylfluoride; DTT: Dithiothreitol; IPG: Immobilized pH Gradient Buffer Solution; IEF: Isoelectric Focusing; PMF: Peptide Mass Fingerprinting.

## Competing interests

The authors declare that they have no competing interests.

## Authors’ contributions

LPZ, CW and ZXK carried out sample collection and protein extraction, WHL and YYM carried out 2-DE, image acquisition and data analysis. LAY and WXD helped in manuscript revision. BQY and DL conceived, designed and implemented this study. All authors read and approved the final manuscript.

## Supplementary Material

Additional file 1: Table S1 The primers used for amplification of target and 16S rRNA genes. **Table S2.** Annealing and extension temperature for amplification of the target and 16S rRNA genes. **Table S3.** (A) The ImageMaster 2D analytical results of differential protein spots (>3-fold) in the gels between 0.5 M NaCl treatment and control (0.02 M). (B) The ImageMaster 2D analytical results of the sole present protein spots in the gels of 0.5 M NaCl treatment or control (0.02 M). (C) The ImageMaster 2D analytical results of differential protein spots (>3-fold) in the gels between 1.0 M NaCl treatment and control (0.02 M). (D) The ImageMaster 2D analytical results of the sole present protein spots in the gels of 1.0 M NaCl treatment or control (0.02 M). (E) The ImageMaster 2D analytical results of differential protein spots (>3-fold) in the gels between 0.5 M NaCl and 1.0 M treatment. (F) The ImageMaster 2D analytical results of the sole present protein spots in the gels of 1.0 M NaCl or 0.5 M treatment. **Table S4.** (A) The MALDI-TOF/MS analytical results of differential protein spots (>3-fold) between 0.5 M NaCl treatment and control (0.02 M). (B) The MS analytical results of the sole present protein spots in 0.5 M NaCl treatment or control (0.02 M). (C) The MS analytical results of differential protein spots (>3-fold) between 1.0 M NaCl treatment and control (0.02 M). (D) The MS analytical results of the sole present protein spots in 1.0 M NaCl treatment or control (0.02 M). (E) The MS analytical results of differential protein spots (>3-fold) between 1.0 M and 0.5 M NaCl treatment. (F) The MS analytical results of the sole present protein spots in 1.0 M NaCl or 0.5 M treatment. **Table S5.** The standard curve equation, regression coefficient and amplified efficiency of the 16S rRNA genes. **Table S6.** The standard curve, regression coefficient and amplified efficiency of the target genes. **Figure S1.** ASP growth curve in different salt concentrations. **Figure S2.** The differential protein profiles of 2-DE of ASP in control (0.02 M), medium salt treatment (0.5 M) and high salt treatment (1.0 M) groups. **Figure S3.** The AGE profile of total RNA in control and medium salt treatment. (Note: Lane 1, control; Lane 2, medium salt treatment.). **Figure S4.** The standard and melting curve of 16S rRNA and sample. Click here for file
