# Supplementary material for: Identification of differentially expressed proteins of Arthrospira (Spirulina) plantensis-YZ under salt-stress conditions by proteomics and qRT-PCR analysis
Source: Proteome Sci. 2013 Jan 30;11:6. doi: 10.1186/1477-5956-11-6 (PMC3599948; doi:10.1186/1477-5956-11-6)
Supplement: Additional file 1: Table S1 — The primers used for amplification of target and 16S rRNA genes. Table S2. Annealing and extension temperature for amplification of the target and 16S rRNA genes. Table S3. (A) The ImageMaster 2D analytical results of differential protein spots (>3-fold) in the gels between 0.5 M NaCl treatment and control (0.02 M). (B) The ImageMaster 2D analytical results of the sole present protein spots in the gels of 0.5 M NaCl treatment or control (0.02 M). (C) The ImageMaster 2D analytical results of differential protein spots (>3-fold) in the gels between 1.0 M NaCl treatment and control (0.02 M). (D) The ImageMaster 2D analytical results of the sole present protein spots in the gels of 1.0 M NaCl treatment or control (0.02 M). (E) The ImageMaster 2D analytical results of differential protein spots (>3-fold) in the gels between 0.5 M NaCl and 1.0 M treatment. (F) The ImageMaster 2D analytical results of the sole present protein spots in the gels of 1.0 M NaCl or 0.5 M treatment. Table S4. (A) The MALDI-TOF/MS analytical results of differential protein spots (>3-fold) between 0.5 M NaCl treatment and control (0.02 M). (B) The MS analytical results of the sole present protein spots in 0.5 M NaCl treatment or control (0.02 M). (C) The MS analytical results of differential protein spots (>3-fold) between 1.0 M NaCl treatment and control (0.02 M). (D) The MS analytical results of the sole present protein spots in 1.0 M NaCl treatment or control (0.02 M). (E) The MS analytical results of differential protein spots (>3-fold) between 1.0 M and 0.5 M NaCl treatment. (F) The MS analytical results of the sole present protein spots in 1.0 M NaCl or 0.5 M treatment. Table S5. The standard curve equation, regression coefficient and amplified efficiency of the 16S rRNA genes. Table S6. The standard curve, regression coefficient and amplified efficiency of the target genes. Figure S1. ASP growth curve in different salt concentrations. Figure S2. The differential protein profiles of 2-DE of [file 1477-5956-11-6-S1.doc]

**Supplementary Table 1**

**The primers used for amplification of target and 16S rRNA** genes

| ORF | Forward primer sequence | Reverse primer sequence | Amplified fragment length |
| --- | --- | --- | --- |
| 3277 | TTCGGAAAGTCCCACATC | CACCCACTAAAGTCCAAGC | 185bp |
| 2281 | GGAATGCGGATGAACCTA | CATCGACAACATAGCCAAA | 201bp |
| 4535 | GTGCGTAAGGTCTATGAGG | CTCTACCAGGTCCCGAAT | 195bp |
| 5055 | TATCTACCCGATGACAAGG | ACGGTGCTAAAGTCTCCC | 215bp |
| 4103 | GGAAATGCCTATACAACCC | CAGGATACCGATACCAACA | 192bp |
| 1911 | TATCGTCCGCACTAACTT | ACATCCCTGTCTTCACAA | 231bp |
| 4633 | ACCTGGATTCTCCTGAGTATTT | GTATTGCGAGCGACTTCC | 193bp |
| 2155 | TCCGTGACTTTATTGGTGG | CCCTTCGGTCCTTTGTTA | 178bp |
| 4030 | ATACGGTTGCTCAATGGC | CGTTCCCGACGATACTCT | 244bp |
| 1989 | GCTATTACACCCGCACAA | TAGCACCAATTACCAAGGAG | 177bp |
| 1251 | CCCAATCATCTCCCTACA | ATGGCGACCACTACCTTC | 197bp |
| 5516 | CAAGTCTGTTTGGCGTAG | GTATTGGGTCCAGTGAGG | 193bp |
| 3023 | GGTAGGCTACACGGAATC | AACCTGTGGCTGAGAAAA | 217bp |
| 3910 | TGACTTGGGATGAGGAGC | ATCTGCACAAGCTGAGGC | 181bp |
| 4938 | TATGCCCTCCAGTTTGCT | GGCGGGTAAGTAGGTTTG | 231bp |
| 3807 | TCCCACTCCTAACGTCTC | CCGTCCGTAATAAAGTCC | 165bp |
| 1688 | CCACCGAAGAAACCACTA | CATTAGCACCCAATCCAC | 258bp |
| 2740 | ATACTTGGCTTTGTTGCG | CCGACAGTTAGGCTCATAC | 252bp |
| 2739 | AAAGCCTCTGCTGTGGAA | CCAGTTGTTGCTGGGAAT | 220bp |
| 1433 | TATCCCGATACCTCAAACC | ATCGCTGAACCAATACCC | 158bp |
| 2287 | ACGGTGAACCCTGATGAA | ACCCTGGAGAACGTGAAT | 240bp |
| 2050 | TGACTGCTGGATTCGCTAC | AGGGCTTTCTCAGGATGG | 225bp |
| 4213 | CTATGACTATTGAGGAGCGTAA | CCATACCGGGAAACTGAC | 177bp |
| 4632 | GTGGTAGTGGCTCCTTCT | AACGACGTACTCTGGGAT | 169bp |
| 4634 | GTTTCTATCGCTGATTCCC | TTAGGTCCCTGCATTTGG | 194bp |
| 4635 | CTGCTCGTTCTTTGTTCG | AAGCCAGGTAAGTTTCACG | 195bp |
| 273 | AACAGCATTTAGTGCGTCAA | CGTCCGAGGTCATTTGTG | 197bp |
| 1198 | GGGTTTAGGGTTTATCAGTC | TACGTCTAGCCGCTTCTT | 197bp |
| 624 | ACAACCGCCTAAGTCCAA | CAAATGCTGTTCCGCCTC | 169bp |
| 16S rRNA | AAGCCTGACGGAGCAAGA | GGACGCTTTACGCCCAAT | 177bp |

**Supplementary Table 2**

**Annealing and extension temperature for amplification of the target and 16S rRNA** genes

| ORF and the corresponding internal standard | T℃ | ORF and the corresponding internal standard | T℃ | ORF and the corresponding internal standard | T℃ |
| --- | --- | --- | --- | --- | --- |
| 3277(16S RNA^12^) | 55.5 | 1251(16S rRNA^1^) | 55.5 | 2287(16S rRNA^8^) | 53.0 |
| 2281(16S rRNA^3^) | 55.5 | 5516(16S rRNA^1^) | 55.5 | 2050(16S rRNA^7^) | 54.0 |
| 4535(16S rRNA^9^) | 54.8 | 3023(16S rRNA^1^) | 55.5 | 4213(16S rRNA^1^) | 55.5 |
| 5055(16S rRNA^10^) | 56.0 | 3910(16S rRNA^6^) | 54.0 | 4632(16S rRNA^11^) | 55.0 |
| 4103(16S rRNA^4^) | 55.0 | 4938(16S rRNA^8^) | 53.0 | 4634(16S rRNA^11^) | 55.0 |
| 1911(16S rRNA^5^) | 55.0 | 3807(16S rRNA^7^) | 54.0 | 4635(16S rRNA^13^) | 54.0 |
| 4633(16S rRNA^11^) | 55.0 | 1688(16S rRNA^14^) | 54.8 | 273(16S rRNA^1^) | 55.5 |
| 2155(16S rRNA^13^) | 54.0 | 2740(16S rRNA^3^) | 55.5 | 1198(16S rRNA^2^) | 54.8 |
| 4030(16S rRNA^5^) | 55.0 | 2739(16S rRNA^9^) | 54.8 | 624(16S rRNA^1^) | 55.5 |
| 1989(16S rRNA^4^) | 55.0 | 1433(16S rRNA^10^) | 54.8 |  |  |

**Supplementary Table 3-A**

**The ImageMaster 2D** analytical results of differential protein spots (>3-fold) in the gels between 0.5 M NaCl treatment and control (0.02 M)

| **SpotID** | **Fold（0.02M）** | **Fold**  **（0.5M）** | **X** | **Y** | **pI** | **MW** | **Area** | **Vol** | **%Vol** | **Intensity** | **%Intensity** |
| --- | --- | --- | --- | --- | --- | --- | --- | --- | --- | --- | --- |
| 94 | -3.07988 | 3.07988 | 1257 | 1943 | 5.98086 | 16929 | 8.93195 | 38761.8 | 0.038879 | 11508 | 0.0594108 |
| 109 | -3.28116 | 3.28116 | 670 | 1861 | 5.04466 | 17970 | 7.21151 | 59244.4 | 0.059424 | 21733.4 | 0.1122 |
| 139 | -3.26907 | 3.26907 | 755 | 1619 | 5.18022 | 21513 | 3.78497 | 16135.9 | 0.016185 | 11890.5 | 0.0613855 |
| 145 | -3.76913 | 3.76913 | 1770 | 1582 | 6.79904 | 22132 | 5.58426 | 30574.5 | 0.030667 | 13659.2 | 0.0705165 |
| 176 | -4.46982 | 4.46982 | 864 | 1438 | 5.35407 | 24719 | 4.65236 | 12038.9 | 0.012075 | 7694.13 | 0.0397215 |
| 199 | -3.03805 | 3.03805 | 767 | 1385 | 5.19936 | 25745 | 6.53767 | 27431.3 | 0.027515 | 11450.3 | 0.0591129 |
| 202 | -3.68118 | 3.68118 | 973 | 1379 | 5.52791 | 25864 | 3.60576 | 10038.1 | 0.010069 | 7248 | 0.0374183 |
| 275 | 3.65037 | -3.65037 | 323 | 1233 | 4.49123 | 28931 | 11.0825 | 158245 | 0.158726 | 24846 | 0.128269 |
| 342 | 3.26159 | -3.26159 | 1074 | 1133 | 5.689 | 31199 | 9.36206 | 91100.9 | 0.091377 | 27038.2 | 0.139587 |
| 398 | -3.0248 | 3.0248 | 1710 | 1043 | 6.70335 | 33050 | 5.08964 | 28070.9 | 0.028156 | 14590 | 0.0753219 |
| 489 | 3.17196 | -3.17196 | 322 | 861 | 4.48963 | 37135 | 6.96061 | 68484.9 | 0.068693 | 24393 | 0.125931 |
| 500 | -3.5566 | 3.5566 | 1795 | 839 | 6.83892 | 37662 | 2.4803 | 5849.49 | 0.005867 | 6239.62 | 0.0322125 |
| 514 | -3.21223 | 3.21223 | 1752 | 817 | 6.77033 | 38196 | 3.13263 | 6491.76 | 0.006511 | 6461.44 | 0.0333576 |
| 526 | -5.04499 | 5.04499 | 1764 | 798 | 6.78947 | 38664 | 2.70252 | 6318.76 | 0.006338 | 6121.2 | 0.0316011 |
| 701 | 4.70026 | -4.70026 | 1578 | 642 | 6.49282 | 42726 | 7.3047 | 68231.2 | 0.068438 | 21333 | 0.11013 |
| 740 | -3.85023 | 3.85023 | 1086 | 594 | 5.70813 | 45116 | 4.67386 | 27459.5 | 0.027543 | 13266.1 | 0.068487 |
| 809 | 3.55088 | -3.55088 | 1373 | 504 | 6.16587 | 50551 | 7.49825 | 54049.4 | 0.054213 | 24055.8 | 0.12419 |
| 922 | -3.13764 | 3.13764 | 451 | 314 | 4.69537 | 64271 | 3.6631 | 12987.4 | 0.013027 | 8193.4 | 0.042299 |

**Supplementary Table 3-B**

**The ImageMaster 2D** analytical results of the sole present protein spots in the gels of 0.5 M NaCl treatment or control (0.02 M)

| **SpotID** | | **X** | **Y** | **pI** | **MW** | **Area** | **Vol** | **%Vol** | **Intensity** | **%Intensity** |
| --- | --- | --- | --- | --- | --- | --- | --- | --- | --- | --- |
| 0.5M | M113 | 644 | 1793 | 4.99249 | 19692 | 9.38357 | 58987.3 | 0.067079 | 18575.1 | 0.0862212 |
|  | M123 | 189 | 1730 | 4.26059 | 20671 | 9.97855 | 70878.5 | 0.080602 | 13592 | 0.0630909 |
|  | M125 | 1184 | 1709 | 5.86113 | 21007 | 7.36922 | 19445.5 | 0.022113 | 7522.11 | 0.0349159 |
|  | M137 | 935 | 1630 | 5.46059 | 22320 | 5.39071 | 19084.8 | 0.021703 | 8272 | 0.0383967 |
|  | M148 | 830 | 1549 | 5.29169 | 23752 | 5.50541 | 29969.2 | 0.03408 | 14652.2 | 0.0680121 |
|  | M166 | 1221 | 1486 | 5.92064 | 24928 | 4.47314 | 21761.1 | 0.024746 | 13416.2 | 0.0622748 |
|  | M175 | 1095 | 1464 | 5.71796 | 25353 | 6.43731 | 29078.5 | 0.033068 | 10510 | 0.0487849 |
|  | M240 | 547 | 1332 | 4.83646 | 28056 | 5.79932 | 21509.8 | 0.024461 | 9411.2 | 0.0436846 |
|  | M243 | 987 | 1329 | 5.54424 | 28121 | 5.0968 | 26293.4 | 0.0299 | 11988 | 0.0556455 |
|  | M252 | 391 | 1317 | 4.58552 | 28381 | 3.93551 | 25156 | 0.028607 | 12931 | 0.0600227 |
|  | M317 | 401 | 1214 | 4.60161 | 30716 | 2.95342 | 14745.1 | 0.016768 | 10380 | 0.0481815 |
|  | M421 | 1664 | 1091 | 6.63324 | 33199 | 4.30827 | 24352.1 | 0.027693 | 13386 | 0.0621347 |
|  | M463 | 1146 | 1013 | 5.8 | 34837 | 7.39072 | 24401.1 | 0.027748 | 9130 | 0.0423793 |
|  | M522 | 1642 | 920 | 6.59786 | 36895 | 4.17924 | 12807 | 0.014564 | 10778.4 | 0.0500308 |
|  | M528 | 1809 | 905 | 6.86649 | 37239 | 2.24374 | 11681.3 | 0.013284 | 12407.5 | 0.0575927 |
|  | M595 | 816 | 804 | 5.26917 | 39635 | 2.20073 | 8160.61 | 0.00928 | 8346 | 0.0387402 |
|  | M657 | 774 | 741 | 5.20161 | 41207 | 3.29034 | 38352.9 | 0.043614 | 21101 | 0.0979459 |
|  | M681 | 1712 | 718 | 6.71046 | 41796 | 2.53765 | 15090.3 | 0.01716 | 12270 | 0.0569545 |
|  | M836 | 853 | 547 | 5.32869 | 49960 | 3.87816 | 23156 | 0.026333 | 15743 | 0.0730753 |
|  | M868 | 1288 | 505 | 6.02842 | 52543 | 4.58784 | 26889 | 0.030578 | 13612 | 0.0631837 |
|  | M966 | 726 | 368 | 5.1244 | 61933 | 2.39428 | 10221.9 | 0.011624 | 12749.9 | 0.059182 |
|  | 1012 | 1033 | 288 | 5.61823 | 69350 | 3.17565 | 22220.4 | 0.025269 | 17680 | 0.0820664 |
| 0.02M | N18 | 1354 | 1237 | 6.13557 | 28842 | 5.01795 | 23307.2 | 0.023378 | 9444 | 0.0487553 |
|  | N65 | 1415 | 2032 | 6.23285 | 15867 | 8.00722 | 37775.5 | 0.03789 | 12712.8 | 0.0656307 |
|  | N156 | 1490 | 1507 | 6.35247 | 23444 | 5.29752 | 12697.2 | 0.012736 | 6195.58 | 0.0319851 |
|  | N184 | 1719 | 1416 | 6.7177 | 25140 | 5.99287 | 13564.9 | 0.013606 | 5970.18 | 0.0308215 |
|  | N210 | 1347 | 1367 | 6.1244 | 26103 | 11.4624 | 43799.9 | 0.043933 | 9564.12 | 0.0493754 |
|  | N251 | 1114 | 1272 | 5.75279 | 28078 | 8.40148 | 88528 | 0.088797 | 32991.1 | 0.170319 |
|  | N283 | 1407 | 1220 | 6.2201 | 29221 | 7.78499 | 49004.9 | 0.049154 | 18580 | 0.0959205 |
|  | N339 | 423 | 1132 | 4.65072 | 31219 | 4.08605 | 27801.3 | 0.027886 | 14921 | 0.0770307 |
|  | N377 | 1350 | 1084 | 6.12919 | 32194 | 3.82081 | 30745.1 | 0.030838 | 16132 | 0.0832825 |
|  | N385 | 806 | 1074 | 5.26156 | 32400 | 6.40864 | 42778.4 | 0.042908 | 16238 | 0.0838298 |
|  | N423 | 1451 | 1002 | 6.29027 | 33929 | 6.12907 | 19269.3 | 0.019328 | 8078 | 0.0417032 |
|  | N424 | 1322 | 998 | 6.08453 | 34016 | 4.50182 | 23349.2 | 0.02342 | 11282 | 0.0582441 |
|  | N555 | 199 | 764 | 4.29346 | 39515 | 3.37636 | 19355.8 | 0.019415 | 10497 | 0.0541915 |
|  | N805 | 311 | 503 | 4.47209 | 50615 | 4.75272 | 55476.2 | 0.055645 | 23385 | 0.120727 |
|  | N928 | 1113 | 308 | 5.7512 | 64761 | 6.7599 | 50970.7 | 0.051125 | 21327 | 0.110102 |
|  | N934 | 1400 | 301 | 6.20893 | 65336 | 5.3477 | 51657 | 0.051814 | 31594.1 | 0.163107 |

**Supplementary Table 3-C**

**The ImageMaster 2D** analytical results of differential protein spots (>3-fold) in the gels between 1.0 M NaCl treatment and control (0.02 M)

| **SpotID** | **Fold（0.02M）** | **Fold**  **（1.0M）** | **X** | **Y** | **pI** | **MW** | **Area** | **Vol** | **%Vol** | **Intensity** | **%Intensity** |
| --- | --- | --- | --- | --- | --- | --- | --- | --- | --- | --- | --- |
| 109 | -4.94399 | 4.94399 | 670 | 1861 | 5.04466 | 17970 | 7.21151 | 59244.4 | 0.059424 | 21733.4 | 0.1122 |
| 128 | -5.06865 | 5.06865 | 751 | 1723 | 5.17384 | 19869 | 4.5735 | 25400.7 | 0.025478 | 14560.6 | 0.0751701 |
| 141 | -3.578 | 3.578 | 710 | 1607 | 5.10845 | 21712 | 5.19716 | 31578 | 0.031674 | 16633.3 | 0.0858705 |
| 153 | -3.12428 | 3.12428 | 537 | 1525 | 4.83254 | 23122 | 7.53409 | 118671 | 0.119031 | 33348.2 | 0.172162 |
| 163 | -6.19807 | 6.19807 | 760 | 1473 | 5.1882 | 24064 | 7.82083 | 19187.3 | 0.019246 | 6236 | 0.0321938 |
| 186 | -3.09544 | 3.09544 | 1112 | 1407 | 5.7496 | 25314 | 4.17207 | 34234.4 | 0.034338 | 21366.2 | 0.110304 |
| 202 | -4.70475 | 4.70475 | 973 | 1379 | 5.52791 | 25864 | 3.60576 | 10038.1 | 0.010069 | 7248 | 0.0374183 |
| 203 | -3.65039 | 3.65039 | 426 | 1377 | 4.6555 | 25904 | 6.55201 | 62416.5 | 0.062606 | 22251 | 0.114872 |
| 209 | -3.87342 | 3.87342 | 786 | 1366 | 5.22967 | 26123 | 4.50899 | 34865.4 | 0.034971 | 20041.7 | 0.103467 |
| 221 | -3.1215 | 3.1215 | 1297 | 1333 | 6.04466 | 26793 | 7.39789 | 50257.3 | 0.05041 | 19025.2 | 0.0982189 |
| 242 | -5.38196 | 5.38196 | 1620 | 1289 | 6.55981 | 27714 | 7.21151 | 20839.4 | 0.020903 | 6650 | 0.0343311 |
| 467 | -3.09146 | 3.09146 | 391 | 926 | 4.59968 | 35621 | 3.80647 | 27029.1 | 0.027111 | 14448 | 0.0745888 |
| 517 | -4.04169 | 4.04169 | 1672 | 805 | 6.64274 | 38491 | 2.85306 | 13323.1 | 0.013364 | 11920 | 0.0615378 |
| 563 | 5.91222 | -5.91222 | 1473 | 761 | 6.32536 | 39591 | 4.0502 | 33394.3 | 0.033496 | 21430.6 | 0.110637 |

**Supplementary Table 3-D**

**The ImageMaster 2D analytical results of the sole present protein spots in the gels of 1.0 M NaCl treatment or control (0.02 M)**

| **SpotID** | | **X** | **Y** | **pI** | **MW** | **Area** | **Vol** | **%Vol** | **Intensity** | **%Intensity** |
| --- | --- | --- | --- | --- | --- | --- | --- | --- | --- | --- |
| 1.0 M | 69 | 1181 | 1804 | 5.89832 | 19259 | 10.9391 | 35207 | 0.047958 | 9061.21 | 0.06314 |
|  | 91 | 702 | 1559 | 5.12115 | 23134 | 7.19717 | 38758.1 | 0.052796 | 15392.6 | 0.107258 |
|  | 96 | 889 | 1519 | 5.42455 | 23839 | 7.49825 | 39186.4 | 0.053379 | 10838 | 0.075521 |
|  | 115 | 1032 | 1454 | 5.65657 | 25031 | 8.24378 | 37803.5 | 0.051495 | 8158 | 0.0568463 |
|  | 352 | 1167 | 915 | 5.87561 | 36328 | 5.04662 | 87157.4 | 0.118724 | 41924.5 | 0.292137 |
|  | 372 | 281 | 850 | 4.43807 | 37833 | 3.94268 | 25644.3 | 0.034932 | 17325 | 0.120723 |
|  | 740 | 575 | 249 | 4.91509 | 70958 | 2.25091 | 18599.4 | 0.025336 | 24151 | 0.168288 |
| 0.02M | 18 | 1354 | 1237 | 6.13557 | 28842 | 5.01795 | 23307.2 | 0.023378 | 9444 | 0.0487553 |
|  | 23 | 118 | 1783 | 4.16427 | 19020 | 4.43013 | 39222.9 | 0.039342 | 22402 | 0.115652 |
|  | 47 | 1106 | 2127 | 5.74003 | 14807 | 7.84951 | 31432.6 | 0.031528 | 8312 | 0.0429113 |
|  | 57 | 1558 | 2068 | 6.46093 | 15457 | 7.71331 | 32381.6 | 0.03248 | 10216 | 0.0527408 |
|  | 61 | 218 | 2060 | 4.32376 | 15547 | 10.2725 | 56147 | 0.056317 | 14006 | 0.0723069 |
|  | 63 | 666 | 2047 | 5.03828 | 15695 | 11.9857 | 144746 | 0.145185 | 20185 | 0.104206 |
|  | 78 | 1360 | 1994 | 6.14514 | 16312 | 11.8925 | 40274.1 | 0.040396 | 7594 | 0.0392045 |
|  | 89 | 83 | 1963 | 4.10845 | 16685 | 5.95702 | 22902.9 | 0.022972 | 9392 | 0.0484868 |
|  | 98 | 1585 | 1922 | 6.50399 | 17190 | 9.29038 | 32578.7 | 0.032678 | 10338.8 | 0.0533747 |
|  | 129 | 661 | 1707 | 5.0303 | 20108 | 5.72046 | 33864.8 | 0.033968 | 14638 | 0.0755697 |
|  | 137 | 921 | 1630 | 5.44498 | 21332 | 4.40863 | 17195.2 | 0.017247 | 11234.9 | 0.0580009 |
|  | 139 | 755 | 1619 | 5.18022 | 21513 | 3.78497 | 16135.9 | 0.016185 | 11890.5 | 0.0613855 |
|  | 176 | 864 | 1438 | 5.35407 | 24719 | 4.65236 | 12038.9 | 0.012075 | 7694.13 | 0.0397215 |
|  | 184 | 1719 | 1416 | 6.7177 | 25140 | 5.99287 | 13564.9 | 0.013606 | 5970.18 | 0.0308215 |
|  | 210 | 1347 | 1367 | 6.1244 | 26103 | 11.4624 | 43799.9 | 0.043933 | 9564.12 | 0.0493754 |
|  | 266 | 383 | 1249 | 4.58692 | 28578 | 4.72404 | 51444.8 | 0.051601 | 20428 | 0.105461 |
|  | 283 | 1407 | 1220 | 6.2201 | 29221 | 7.78499 | 49004.9 | 0.049154 | 18580 | 0.0959205 |
|  | 339 | 423 | 1132 | 4.65072 | 31219 | 4.08605 | 27801.3 | 0.027886 | 14921 | 0.0770307 |
|  | 377 | 1350 | 1084 | 6.12919 | 32194 | 3.82081 | 30745.1 | 0.030838 | 16132 | 0.0832825 |
|  | 385 | 806 | 1074 | 5.26156 | 32400 | 6.40864 | 42778.4 | 0.042908 | 16238 | 0.0838298 |
|  | 410 | 1523 | 1024 | 6.4051 | 33455 | 5.17566 | 14825.8 | 0.014871 | 7390 | 0.0381514 |
|  | 423 | 1451 | 1002 | 6.29027 | 33929 | 6.12907 | 19269.3 | 0.019328 | 8078 | 0.0417032 |
|  | 424 | 1322 | 998 | 6.08453 | 34016 | 4.50182 | 23349.2 | 0.02342 | 11282 | 0.0582441 |
|  | 461 | 1363 | 942 | 6.14992 | 35258 | 4.90325 | 24896.1 | 0.024972 | 14226 | 0.0734427 |
|  | 747 | 1128 | 573 | 5.77512 | 46329 | 4.15056 | 20913.8 | 0.020977 | 12424 | 0.0641397 |
|  | 784 | 1319 | 525 | 6.07974 | 49227 | 8.07173 | 29848.2 | 0.029939 | 11330 | 0.0584919 |
|  | 857 | 1354 | 421 | 6.13557 | 56142 | 2.9821 | 14398.6 | 0.014442 | 13320.9 | 0.06877 |
|  | 907 | 1370 | 334 | 6.16108 | 62667 | 2.02869 | 8241.48 | 0.008266 | 11259.5 | 0.0581279 |
|  | 928 | 1113 | 308 | 5.7512 | 64761 | 6.7599 | 50970.7 | 0.051125 | 21327 | 0.110102 |
|  | 967 | 899 | 191 | 5.40989 | 79364 | 4.1864 | 11198.6 | 0.011233 | 6842 | 0.0353223 |

**Supplementary Table 3-E**

**The ImageMaster 2D analytical results of differential protein spots (>3-fold) in the gels between 0.5 M NaCl and 1.0M treatment**

| **SpotID** | **Fold（0.5M）** | **Fold**  **（1.0M）** | **X** | **Y** | **pI** | **MW** | **Area** | **Vol** | **%Vol** | **Intensity** | **%Intensity** |
| --- | --- | --- | --- | --- | --- | --- | --- | --- | --- | --- | --- |
| 344 | -3.24492 | 3.24492 | 864 | 1188 | 5.34638 | 31269 | 3.2545 | 32652.6 | 0.037132 | 21549 | 0.100025 |
| 542 | -5.01986 | 5.01986 | 427 | 865 | 4.64343 | 38170 | 3.35486 | 19043.8 | 0.021656 | 11219 | 0.052076 |
| 565 | -4.29392 | 4.29392 | 678 | 831 | 5.04718 | 38980 | 2.73837 | 17165.8 | 0.019521 | 10769 | 0.0499872 |
| 747 | -3.12179 | 3.12179 | 660 | 655 | 5.01823 | 43886 | 5.30469 | 14835.9 | 0.016871 | 7663 | 0.0355698 |
| 778 | 3.26455 | -3.26455 | 1264 | 624 | 5.98981 | 45550 | 3.48389 | 40294.6 | 0.045822 | 28151 | 0.13067 |
| 800 | -3.57163 | 3.57163 | 317 | 596 | 4.46649 | 47107 | 1.88532 | 7858.01 | 0.008936 | 8752 | 0.0406247 |
| 839 | -3.98922 | 3.98922 | 810 | 540 | 5.25952 | 50381 | 2.81722 | 34169.6 | 0.038857 | 23375 | 0.108501 |
| 908 | -3.65436 | 3.65436 | 1006 | 436 | 5.5748 | 57079 | 2.00001 | 26184.5 | 0.029777 | 29774.1 | 0.138204 |

**Supplementary Table 3-F**

**The ImageMaster 2D analytical results of the sole present protein spots in the gels of 1.0 M NaCl or 0.5M treatment**

| **SpotID** | | **X** | **Y** | **pI** | **MW** | **Area** | **Vol** | **%Vol** | **Intensity** | **%Intensity** |
| --- | --- | --- | --- | --- | --- | --- | --- | --- | --- | --- |
| 1.0 M | 69 | 1181 | 1804 | 5.89832 | 19259 | 10.9391 | 35207 | 0.047958 | 9061.21 | 0.06314 |
|  | 91 | 702 | 1559 | 5.12115 | 23134 | 7.19717 | 38758.1 | 0.052796 | 15392.6 | 0.107258 |
|  | 138 | 419 | 1388 | 4.66198 | 26302 | 15.4123 | 167772 | 0.228536 | 23547 | 0.164079 |
|  | 185 | 917 | 1278 | 5.46998 | 28565 | 7.62012 | 42523.6 | 0.057925 | 14675 | 0.102258 |
|  | 372 | 281 | 850 | 4.43807 | 37833 | 3.94268 | 25644.3 | 0.034932 | 17325 | 0.120723 |
|  | 441 | 1430 | 755 | 6.30233 | 40146 | 4.10755 | 39499.6 | 0.053806 | 15526 | 0.108188 |
| 0.5M | 8 | 1181 | 1804 | 5.89832 | 19259 | 10.9391 | 35207 | 0.047958 | 9061.21 | 0.06314 |
|  | 42 | 702 | 1559 | 5.12115 | 23134 | 7.19717 | 38758.1 | 0.052796 | 15392.6 | 0.107258 |
|  | 78 | 419 | 1388 | 4.66198 | 26302 | 15.4123 | 167772 | 0.228536 | 23547 | 0.164079 |
|  | 109 | 917 | 1278 | 5.46998 | 28565 | 7.62012 | 42523.6 | 0.057925 | 14675 | 0.102258 |
|  | 111 | 281 | 850 | 4.43807 | 37833 | 3.94268 | 25644.3 | 0.034932 | 17325 | 0.120723 |
|  | 113 | 1430 | 755 | 6.30233 | 40146 | 4.10755 | 39499.6 | 0.053806 | 15526 | 0.108188 |
|  | 125 | 1622 | 740 | 6.56568 | 41232 | 2.23657 | 29963.5 | 0.034074 | 20255 | 0.0940189 |
|  | 134 | 254 | 2077 | 4.36515 | 15792 | 8.60937 | 47536.4 | 0.054058 | 14374 | 0.0667207 |
|  | 137 | 1267 | 1965 | 5.99464 | 17228 | 10.7312 | 105300 | 0.119744 | 21264 | 0.0987025 |
|  | 178 | 353 | 1812 | 4.5244 | 19403 | 8.78858 | 133748 | 0.152096 | 23439 | 0.108798 |
|  | 184 | 166 | 1807 | 4.22359 | 19479 | 5.43372 | 25355.6 | 0.028834 | 13041.4 | 0.0605351 |
|  | 290 | 644 | 1793 | 4.99249 | 19692 | 9.38357 | 58987.3 | 0.067079 | 18575.1 | 0.0862212 |
|  | 302 | 1184 | 1709 | 5.86113 | 21007 | 7.36922 | 19445.5 | 0.022113 | 7522.11 | 0.0349159 |
|  | 313 | 798 | 1641 | 5.24021 | 22132 | 6.67387 | 46526.7 | 0.052909 | 20077.2 | 0.0931936 |
|  | 361 | 935 | 1630 | 5.46059 | 22320 | 5.39071 | 19084.8 | 0.021703 | 8272 | 0.0383967 |
|  | 369 | 912 | 1450 | 5.42359 | 25627 | 7.02513 | 47463.8 | 0.053975 | 17346.1 | 0.0805165 |
|  | 371 | 1287 | 1439 | 6.02681 | 25844 | 10.7528 | 78314.9 | 0.089058 | 19318.6 | 0.0896724 |
|  | 384 | 1674 | 1260 | 6.64933 | 29650 | 7.46241 | 37578.1 | 0.042733 | 12708 | 0.0589875 |

**Supplementary Table 4-A**

**The MALDI-TOF/MS** analytical results of differential protein spots (>3-fold) between 0.5 M NaCl treatment and control (0.02 M)

| Spot(0.02M) | Spot(0.5M) | ORF | Accession NO. | Gene Product (best hit in NCBInr database) | Theoretical/Experimental | | | Matched Peptides | Cov | MASCOT Score | E-value |
| --- | --- | --- | --- | --- | --- | --- | --- | --- | --- | --- | --- |
|  |  |  |  |  | MW | pI | |  |  |  |  |
| **Carbohydrate transport and metabolism** | | | |  |  |  | |  |  |  |  |
| N489 | 542 | ORF3807 | ZP_03274253 | Glyceraldehyde-3-phosphate Dehydrogenase, type I [*Arthrospira maxima* CS-328] | 36534/37135 | 6.07/4.48 | | 12 | 52% | 135 | 3.3E-08 |
| **Cell envelope biogenesis, outer membrane** | | | |  |  |  | |  |  |  |  |
| N139 | 134 | ORF4043 | ZP_03274588 | dTDP-4-dehydrorhamnose 3,5-epimerase [*Arthrospira maxima* CS-328] | 20785/22132 | 5.00/5.24 | | 13 | 70% | 138 | 1.7E-08 |
| N809 | 858 | ORF3402 | ZP_03272127 | Glycoside hydrolase family 25 [*Arthrospira maxima* CS-328] | 50658/50551 | 5.22/6.16 | | 10 | 20% | 88 | 0.0015 |
| **Cell motility and secretion / Intracellular trafficking and secretion** | | | | |  |  | |  |  |  |  |
| N526 | 582 | ORF3769 | ZP_06381070 | Twitching motility protein [*Arthrospira* str. Paraca] | 40645/38664 | 8.22/6.78 | | 25 | 51% | 152 | 6.6e-10 |
| **Coenzyme metabolism** | |  |  |  |  |  | |  |  |  |  |
| N202 | 198 | ORF4488 | YP_001516744 | Pterin-4-alpha-carbinolamine dehydratase *[Acaryochloris marina* MBIC11017] | 10179/25864 | 5.42/5.52 | | 7 | 59% | 80 | 0.0096 |
| **Energy production and conversion** | | | |  |  |  | |  |  |  |  |
| N109 | 93 | ORF2739 | ZP_03274295 | ATP synthase F0, B subunit [*Arthrospira maxima* CS-328] | 19538/17970 | 5.14/5.04 | | 13 | 59% | 114 | 4.2e-06 |
|  |  |  |  |  |  |  | |  |  |  |  |
| Spot(0.02M) | Spot(0.5M) | ORF | Accession NO. | Gene Product (best hit in NCBInr database) | Theoretical/Experimental | | | Matched Peptides | Cov | MASCOT Score | E-value |
|  |  |  |  |  | MW | | pI |  |  |  |  |
| N145 | M140 | ORF2740 | ZP_06381970 | F0F1 ATP synthase subunit delta [*Arthrospira* str. Paraca] | 20108/22132 | 6.15/6.79 | | 15 | 53% | 116 | 2.6e-06 |
| **General function prediction only** | | |  |  |  |  | |  |  |  |  |
| N701 | M718 | ORF3277 | ZP_03275759 | FAD-dependent pyridine nucleotide-disulphide oxidoreductase [*Arthrospira maxima* CS-328] | 47147/42726 | 6.67/6.49 | | 6 | 20% | 78 | 0.018 |
| **Posttranslational modification, protein turnover, chaperones** | | | | |  |  | |  |  |  |  |
| N275 | M305 | ORF2281 | ZP_06380867 | Peptidyl-prolyl cis-trans isomerase, cyclophilin type [*Arthrospira* str. Paraca] | 23921/28931 | 4.74/4.49 | | 6 | 36% | 75 | 0.036 |
| N922 | M1011 | ORF1976 | ZP_03275215 | Chaperonin GroEL [*Arthrospira maxima* CS-328] | 58209/64271 | 5.00/4.69 | | 32 | 56% | 190 | 1e-13 |
| **Signal transduction mechanisms** | | | |  |  |  | |  |  |  |  |
| N199 | M196 | ORF2861 | ZP_06382415 | Stress protein [*Arthrospira* str. Paraca] | 22195/25745 | 4.93/5.19 | | 23 | 93% | 148 | 1.7e-09 |
| **Function unknown** | |  |  |  |  |  | |  |  |  |  |
| N94 | M78 | ORF4634 | ZP_03271568 | Phycocyanin, alpha subunit [*Arthrospira maxima* CS-328] | 17703/17228 | 5.82/5.99 | | 11 | 74% | 78 | 0.015 |
| N176 | M178 | ORF1456 | ZP_06380822 | Pentapeptide repeat-containing protein [*Arthrospira* str. Paraca] | 19860/25627 | 5.13/5.42 | | 12 | 47% | 77 | 0.021 |
| N342 | M394 | ORF2155 | ZP_06382427 | Phycobilisome linker polypeptide [*Arthrospira* str. Paraca] | 29450/31199 | 9.25/5.68 | | 15 | 55% | 218 | 1.6E-16 |
| N398 | M435 | ORF2155 | ZP_06382427 | Phycobilisome linker polypeptide [*Arthrospira* str. Paraca] | 29450/33050 | 9.25/6.70 | | 30 | 71% | 269 | 1.3e-21 |
| N500 | M548 | ORF4030 | ZP_03276569 | Gas vesicle protein GvpC [*Arthrospira maxima* CS-328] | 32452/37662 | 9.22/6.83 | | 39 | 76% | 310 | 1e-25 |
| Spot(0.02M) | Spot(0.5M) | ORF | Accession NO. | Gene Product (best hit in NCBInr database) | Theoretical/Experimental | | | Matched Peptides | Cov | MASCOT Score | E-value |
|  |  |  |  |  | MW | | pI |  |  |  |  |
| N514 | M560 | ORF4004 | ZP_06381784 | Peptidoglycan binding domain-containing protein [*Arthrospira* str. Paraca] | 39737/38196 | 6.32/6.77 | | 17 | 46% | 196 | 2.6E-14 |
| N740 | M813 | ORF2313 | ZP_06384820 | Type III effector Hrp-dependent outers [*Arthrospira* str. Paraca] | 34715/45116 | 4.97/5.70 | | 16 | 56% | 116 | 2.6e-05 |

**Supplementary Table 4-B**

**The MS analytical results of the sole present protein spots in 0.5 M NaCl treatment or control (0.02 M)**

| Spot(0.02M) | Spot(0.5M) | ORF | Accession NO. | Gene Product (best hit in ncbinr database) | Theoretical/experimental | | Matched Peptides | Cov | MASCOT Score | E-value |
| --- | --- | --- | --- | --- | --- | --- | --- | --- | --- | --- |
|  |  |  |  |  | MW | pI |  |  |  |  |
| **Amino acid transport and metabolism / Cell envelope biogenesis, outer membrane** | | | | |  |  |  |  |  |  |
|  | M463 | ORF3910 | ZP_06382240 | Dihydrodipicolinate synthase [*Arthrospira* str. Paraca] | 30869/34837 | 5.20/5.80 | 22 | 51% | 141 | 8.3e-09 |
| **Carbohydrate transport and metabolism** | | | |  |  |  |  |  |  |  |
|  | M595 | ORF3807 | ZP_03274253 | Glyceraldehyde-3-phosphate dehydrogenase, type I [*Arthrospira maxima* CS-328] | 36534/39635 | 6.07/5.26 | 24 | 61% | 110 | 0.00001 |
|  | M681 | ORF3807 | ZP_03274253 | Glyceraldehyde-3-phosphate dehydrogenase, type I [*Arthrospira maxima* CS-328] | 36534/41796 | 6.07/6.71 | 23 | 54% | 164 | 4.2e-11 |
|  | M966 | ORF1396 | ZP_06380414 | Phosphoglucomutase [*Arthrospira* str. Paraca] | 59997/61933 | 5.05/5.12 | 17 | 43% | 93 | 0.0005 |
| N928 |  | ORF4691 | ZP_06382051 | Transketolase [*Arthrospira* str. Paraca] | 72979/64761 | 5.78/5.75 | 21 | 45% | 204 | 4.1E-15 |
| **Cell envelope biogenesis, outer membrane** | | | |  |  |  |  |  |  |  |
|  | M522 | ORF5367 | ZP_06382090 | Glycosyl transferase family protein [*Arthrospira* str. Paraca] | 35242/36895 | 5.98/6.59 | 10 | 31% | 76 | 0.023 |
| **Cell envelope biogenesis, outer membrane / Carbohydrate transport and metabolism** | | | | |  |  |  |  |  |  |
|  | M528 | ORF0323 | ZP_06381275 | Dtdp-glucose 4,6-dehydratase [*Arthrospira* str. Paraca] | 35782/37239 | 6.08/6.86 | 20 | 58% | 140 | 1e-08 |
| N251 |  | ORF1751 | ZP_06381424 | NAD-dependent epimerase/dehydratase [*Arthrospira* str. Paraca] | 23630/28078 | 5.15/5.75 | 12 | 70% | 184 | 4.1E-13 |
|  |  |  |  |  |  | |  |  |  |  |
| Spot(0.02M) | Spot(0.5M) | ORF | Accession NO. | Gene Product (best hit in ncbinr database) | Theoretical/experimental | | Matched Peptides | Cov | MASCOT Score | E-value |
|  |  |  |  |  | MW | pI |  |  |  |  |
| **Cell division and chromosome partitioning** | | | |  |  |  |  |  |  |  |
|  | M125 | ORF0639 | ZP_02928939 | FHA domain containing protein [*Verrucomicrobium spinosum* DSM 4136] | 170844/21007 | 5.09/5.86 | 27 | 19% | 84 | 0.0041 |
| **Energy production and conversion** | | | |  |  |  |  |  |  |  |
|  | M371 | ORF2754 | ZP_06385149 | NADH dehydrogenase subunit B [*Arthrospira* str. Paraca] | 27251/32011 | 6.75/6.69 | 16 | 48% | 191 | 8.3e-14 |
|  | M868 | ORF3397 | ZP_06382626 | Aldehyde dehydrogenase [*Arthrospira* str. Paraca] | 48847/52543 | 5.68/6.02 | 16 | 36% | 131 | 8.3E-08 |
| **General function prediction only** | | |  |  |  |  |  |  |  |  |
|  | M317 |  | ZP_03130156 | Conserved hypothetical protein [C*hthoniobacter flavus* Ellin428] | 73654/30716 | 6.98/4.60 | 17 | 27% | 77 | 0.019 |
|  | M1012 | ORF3860 | ZP_06382439 | Carbonate dehydratase [*Arthrospira* str. Paraca] | 59145/69350 | 8.66/5.61 | 27 | 47% | 197 | 2.1E-14 |
| N934 |  | ORF1251 | ZP_06381540 | Hypothetical protein aplap_07632 [*Arthrospira* str. Paraca] | 67407/65336 | 5.66/6.20 | 19 | 42% | 166 | 2.6E-11 |
| **Posttranslational modification, protein turnover, chaperones** | | | | |  |  |  |  |  |  |
|  | M113 | ORF1746 | ZP_06383547 | Alkyl hydroperoxide reductase/ Thiol specific antioxidant/ Mal allergen [*Arthrospira* str. Paraca] | 16988/19692 | 5.07/4.99 | 8 | 71% | 108 | 1.7e-05 |
|  | M123 | ORF1368 | ZP_03273475 | Redoxin domain protein [*Arthrospira maxima* CS-328] | 19780/20671 | 4.88/4.26 | 10 | 52% | 87 | 0.002 |
| N156 |  | ORF1368 | ZP_03273475 | Redoxin domain protein [*Arthrospira maxima* CS-328] | 19780/23444 | 4.88/6.35 | 10 | 49% | 125 | 3.3E-07 |
|  |  |  |  |  |  |  |  |  |  |  |
| N805 |  | ORF1434 | ZP_06380920 | Chaperonin groel [*Arthrospira* str. Paraca] | 57425/50615 | 5.00/4.47 | 24 | 50% | 271 | 8.2E-22 |
| **Inorganic ion transport and metabolism** | | | |  |  |  |  |  |  |  |
|  | M137 | ORF1911 | ZP_06383116 | Adenylylsulfate kinase [*Arthrospira* str. Paraca] | 19897/22320 | 5.22/5.46 | 16 | 79% | 200 | 1e-14 |
| Spot(0.02M) | Spot(0.5M) | ORF | Accession NO. | Gene Product (best hit in ncbinr database) | Theoretical/experimental | | Matched Peptides | Cov | MASCOT Score | E-value |
|  |  |  |  |  | MW | pI |  |  |  |  |
| **Nucleotide transport and metabolism** | | | |  |  |  |  |  |  |  |
|  | M148 | ORF3887 | ZP_06380517 | Bifunctional pyrimidine regulatory protein pyrr uracil phosphoribosyltransferase [*Arthrospira* str. Paraca] | 19867/23752 | 5.10/5.29 | 22 | 92% | 227 | 2.1e-17 |
|  | M243 |  | ZP_03275636 | Adenylate kinase [*Arthrospira maxima* CS-328] | 21863/28121 | 5.22/5.54 | 14 | 82% | 94 | 0.00043 |
| **Signal transduction mechanisms** | | |  |  |  |  |  |  |  |  |
| N555 |  | ORF1869 | YP_002485086 | Multi-sensor signal transduction histidine kinase [*Cyanothece* sp. PCC 7425] | 85539/39515 | 5.95/4.29 | 11 | 12% | 74 | 0.044 |
| **Translation, ribosomal structure and biogenesis** | | | |  |  |  |  |  |  |  |
|  | M166 | ORF5117 | ZP_06381795 | Ribosome recycling factor [*Arthrospira* str. Paraca] | 20263/24928 | 5.71/5.92 | 12 | 63% | 145 | 3.3e-09 |
|  | M657 |  | ZP_05756741 | RNA polymerase ECF-type sigma factor [*Bacteroides* sp. D2] | 10349/41207 | 6.26/5.20 | 8 | 98% | 84 | 0.0042 |
|  | M836 | ORF3535 | ZP_06385129 | Elongation factor Tu [*Arthrospira* str. Paraca] | 42538/49960 | 5.08/5.32 | 33 | 85% | 260 | 1e-20 |
| N210 |  | ORF5117 | ZP_06381795 | Ribosome recycling factor [*Arthrospira* str. Paraca] | 20263/26103 | 5.71/6.12 | 10 | 59% | 140 | 1E-08 |
| N283 |  | ORF4662 | ZP_03271593 | Sigma 54 modulation protein/ribosomal protein S30EA [*Arthrospira maxima* CS-328] | 23973/29221 | 7.03/6.22 | 9 | 58% | 94 | 0.00044 |
| N377 |  | ORF1074 | ZP_06380559 | Elongation factor TS [*Arthrospira* str. Paraca] | 24626/32194 | 5.74/6.12 | 7 | 53% | 75 | 0.034 |
| **Function unknown** | |  |  |  |  |  |  |  |  |  |
|  | M175 | ORF0273 | ZP_06384609 | Hypothetical protein aplap_23383 [*Arthrospira* str. Paraca] | 20229/25353 | 5.93/5.71 | 9 | 49% | 121 | 8.3e-07 |
|  | M240 |  |  |  |  |  |  |  |  |  |
|  | M252 | ORF4635 | ABD64607 | Phycocyanin beta chain [*Arthrospira*] | 18506/28381 | 5.19/4.58 | 11 | 55% | 98 | 0.00016 |
| Spot(0.02M) | Spot(0.5M) | ORF | Accession NO. | Gene Product (best hit in ncbinr database) | Theoretical/experimental | | Matched Peptides | Cov | MASCOT Score | E-value |
|  |  |  |  |  | MW | pI |  |  |  |  |
|  | M421 | ORF2155 | ZP_06382427 | Phycobilisome linker polypeptide [*Arthrospira* str. Paraca] | 29450/33199 | 9.25/6.63 | 34 | 83% | 340 | 1e-28 |
| N18 |  |  | YP_003289399 | Hypothetical protein Rmar_0102 [*Rhodothermus marinus* DSM 4252] | 25840/28842 | 7.88/6.13 | 7 | 31% | 74 | 0.04 |
| N65 |  | ORF4030 | ZP_06384870 | Hypothetical protein aplap_24737 [*Arthrospira* str. Paraca] | 17324/15867 | 5.96/6.23 | 18 | 86% | 245 | 3.3E-19 |
| N184 |  |  |  |  |  |  |  |  |  |  |
| N339 |  | ORF0361 | ZP_06384062 | Peptidase S8 and S53 subtilisin kexin sedolisin [*Arthrospira* str. Paraca] | 44238/31219 | 4.59/4.65 | 16 | 44% | 200 | 1E-14 |
| N385 |  | ORF2155 | ZP_06382427 | Phycobilisome linker polypeptide [*Arthrospira* str. Paraca] | 29450/32400 | 9.25/5.26 | 23 | 67% | 269 | 1.3E-21 |
| N423 |  | ORF4633 | ABV01983 | Cpch [*Arthrospira* Sp-16] | 30852/33929 | 7.82/6.29 | 25 | 60% | 295 | 3.3E-24 |
| N424 |  | ORF5516 | ZP_03272395 | Conserved hypothetical protein [*Arthrospira maxima* CS-328] | 41116/34016 | 6.04/6.08 | 13 | 50% | 205 | 3.3E-15 |

**Supplementary Table 4-C**

**The MS analytical results of differential protein spots (>3-fold) between 1.0 M NaCl treatment and control (0.02 M)**

| Spot(0.02M) | Spot(1.0M) | ORF | Accession NO. | Gene Product (best hit in ncbinr database) | Theoretical/Experimental | | Matched Peptides | Cov | MASCOT Score | E-value |
| --- | --- | --- | --- | --- | --- | --- | --- | --- | --- | --- |
|  |  |  |  |  | MW | pI |  |  |  |  |
| **Carbohydrate transport and metabolism** | | | |  |  |  |  |  |  |  |
| N563 | H425 | ORF0842 | ZP_06383392 | Fructose 1,6-bisphosphatase II [*Arthrospira* str. Paraca] | 37477/39591 | 5.26/6.32 | 21 | 51% | 155 | 3.30E-10 |
| **Cell envelope biogenesis, outer membrane** | | | |  |  |  |  |  |  |  |
| N242 | H169 | ORF4201 | ZP_06382028 | Putative D-alanyl-D-alanine dipeptidase [*Arthrospira* str. Paraca] | 27272/27714 | 7.74/6.55 | 19 | 51% | 211 | 8.3e-16 |
| **General function prediction only** | | | |  |  |  |  |  |  |  |
| N186 | H123 |  | ZP_06380648 | Hypothetical protein aplap_03077 [*Arthrospira* str. Paraca] | 24970/25314 | 5.61/5.74 | 12 | 54% | 136 | 2.6e-08 |
| N203 | H138 | ORF1601 | ZP_02733328 | NB-ARC domain protein [*Gemmata obscuriglobus* UQM 2246] | 53287/26302 | 8.98/4.66 | 13 | 24% | 82 | 0.0065 |
| **Posttranslational modification, protein turnover, chaperones** | | | | |  |  |  |  |  |  |
| N141 | H84 | ORF1368 | ZP_03273475 | Redoxin domain protein [*Arthrospira maxima* CS-328] | 19780/21712 | 4.88/5.10 | 24 | 68% | 171 | 8.3e-12 |
| **Inorganic ion transport and metabolism** | | | |  |  |  |  |  |  |  |
| N153 | H98 | ORF4792 | ZP_06382815 | DNA starvation/stationary phase protection protein Dps [*Arthrospira* str. Paraca] | 19666/23122 | 4.93/4.83 | 14 | 70% | 109 | 1.3e-05 |
| **Nucleotide transport and metabolism** | | | |  |  |  |  |  |  |  |
| N209 | H130 |  | ZP_03275636 | Adenylate kinase [*Arthrospira maxima* CS-328] | 21863/26123 | 5.22/5.22 | 13 | 77% | 103 | 5.2e-05 |
| Spot(0.02M) | Spot(1.0M) | ORF | Accession NO. | Gene Product (best hit in ncbinr database) | Theoretical/Experimental | | Matched Peptides | Cov | MASCOT Score | E-value |
|  |  |  |  |  | MW | pI |  |  |  |  |
| **Translation, ribosomal structure and biogenesis** | | | | |  |  |  |  |  |  |
| N163 | H103 | ORF1074 | ZP_06380559 | Elongation factor TS [*Arthrospira* str. Paraca] | 24626/24064 | 5.74/5.18 | 14 | 56% | 113 | 5.2e-06 |
| N517 | H384 | ORF4938 | ZP_06381009 | Glutathione synthetase [*Arthrospira* str. Paraca] | 36159/38491 | 5.93/6.64 | 15 | 43% | 100 | 0.0001 |
| **Function unknown** | |  |  |  |  |  |  |  |  |  |
| N109 | H58 | ORF4634 | ZP_03271568 | Phycocyanin, alpha subunit [*Arthrospira* str. Paraca] | 17719/17970 | 5.82/5.04 | 12 | 75% | 91 | 0.00076 |
| N128 | H75 | ORF4634 | ZP_03271568 | Phycocyanin, alpha subunit [*Arthrospira maxima* CS-328] | 17703/19259 | 5.82/5.89 | 10 | 58% | 91 | 0.00076 |
| N202 | H127 | ORF1198 | ZP_06382670 | Hypothetical protein aplap_13413 [*Arthrospira* str. Paraca] | 29241/25864 | 8.15/5.52 | 11 | 54% | 103 | 5.2e-05 |
| N221 | H149 |  |  |  |  |  |  |  |  |  |
| N467 | H342 | ORF4632 | ABB84420 | Cpci [*Arthrospira* Sp-5] | 32790/35621 | 8.33/4.59 | 19 | 53% | 150 | 1.00E-09 |

**Supplementary Table 4-D**

**The MS analytical results of the sole present protein spots in 1.0 M NaCl treatment or control (0.02 M)**

| Spot(0.02M) | Spot(1.0M) | ORF | Accession NO. | Gene Product (best hit in ncbinr database) | Theoretical/Experimental | | Matched Peptides | Cov | MASCOT Score | E-value |
| --- | --- | --- | --- | --- | --- | --- | --- | --- | --- | --- |
|  |  |  |  |  | MW（kDa） | pI |  |  |  |  |
| **Carbohydrate transport and metabolism** | | | |  |  |  |  |  |  |  |
|  | H740 | ORF5508 | ZP_03272404 | Ribulose-bisphosphate carboxylase [*Arthrospira maxima* CS-328] | 53905/70958 | 6.04/4.91 | 24 | 44% | 131 | 8.3e-08 |
| N747 |  | ORF5508 | ZP_03272404 | Ribulose-bisphosphate carboxylase [*Arthrospira maxima* CS-328] | 53905/46329 | 6.04/5.77 | 25 | 42% | 230 | 1.00E-17 |
| N857 |  | ORF4691 | ZP_06382051 | Transketolase [*Arthrospira* str. Paraca] | 72979/56142 | 5.78/6.13 | 16 | 38% | 174 | 4.10E-12 |
| N907 |  | ORF4691 | ZP_06382051 | Transketolase [*Arthrospira* str. Paraca] | 72979/62667 | 5.78/6.16 | 14 | 36% | 148 | 1.60E-09 |
| N928 |  | ORF4691 | ZP_06382051 | Transketolase [*Arthrospira* str. Paraca] | 72979/64761 | 5.78/5.75 | 21 | 45% | 204 | 4.10E-15 |
| N967 |  | ORF0589 | ZP_06382353 | Phosphoenolpyruvate synthase [*Arthrospira* str. Paraca] | 84399/79364 | 5.41/5.40 | 18 | 28% | 136 | 2.60E-08 |
| **Cell envelope biogenesis, outer membrane / Carbohydrate transport and metabolism** | | | | |  |  |  |  |  |  |
| N461 |  | ORF4535 | ZP_06381408 | NAD-dependent epimerase/dehydratase [*Arthrospira* str. Paraca] | 35652/35258 | 5.59/6.14 | 23 | 53% | 260 | 1.00E-20 |
| **Defense mechanisms** | | | |  |  |  |  |  |  |  |
| N23 |  | ORF3062 | YP_001866475 | Hypothetical protein Npun_F3030 [*Nostoc punctiforme* PCC 73102] | 41898/19020 | 9.91/4.16 | 5 | 18% | 62 | 0.59 |
| **General function prediction only** | | |  |  |  |  |  |  |  |  |
|  | H115 | ORF0370 | ZP_06382005 | Phospholipase/Carboxylesterase [*Arthrospira* str. Paraca] | 23206/25031 | 5.37/5.65 | 10 | 43% | 91 | 0.00083 |
| Spot(0.02M) | Spot(1.0M) | ORF | Accession NO. | Gene Product (best hit in ncbinr database) | Theoretical/Experimental | | Matched Peptides | Cov | MASCOT Score | E-value |
|  |  |  |  |  | MW（kDa） | pI |  |  |  |  |
| **Posttranslational modification, protein turnover, chaperones** | | | | |  |  |  |  |  |  |
|  | H91 | ORF1368 | ZP_03273475 | Redoxin domain protein [*Arthrospira maxima* CS-328] | 19780/23134 | 4.88/5.12 | 11 | 59% | 80 | 0.011 |
| N47 |  | ORF4103 | ZP_03271327 | Allophycocyanin, beta subunit [*Arthrospira maxima* CS-328] | 17433/14807 | 6.26/5.74 | 14 | 84% | 165 | 3.30E-11 |
| N57 |  | ORF4103 | ZP_03271327 | Allophycocyanin, beta subunit [*Arthrospira maxima* CS-328] | 17433/15457 | 6.26/6.4 | 15 | 91% | 154 | 4.10E-10 |
| N63 |  | ORF4103 | ZP_03271327 | Allophycocyanin, beta subunit [*Arthrospira maxima* CS-328] | 17433/15695 | 6.26/5.03 | 15 | 91% | 163 | 5.2E-11 |
| N129 |  | ORF1368 | ZP_03273475 | Redoxin domain protein [*Arthrospira maxima* CS-328] | 19780/20108 | 4.88/5.03 | 10 | 49% | 119 | 1.30E-06 |
| N139 |  | ORF1368 | ZP_03273475 | Redoxin domain protein [*Arthrospira maxima* CS-328] | 19780/21513 | 4.88/5.18 | 13 | 59% | 125 | 3.30E-07 |
| **Inorganic ion transport and metabolism** | | | | |  |  |  |  |  |  |
| N137 |  | ORF1911 | ZP_06383116 | Adenylylsulfate kinase [*Arthrospira* str. Paraca] | 19897/21332 | 5.22/5.44 | 10 | 70% | 152 | 6.50E-10 |
| **Nucleotide transport and metabolism** | | | | |  |  |  |  |  |  |
|  |  |  |  |  |  |  |  |  |  |  |
| N176 |  |  | ZP_03275636 | Adenylate kinase [*Arthrospira maxima* CS-328] | 21863/24719 | 5.22/5.35 | 11 | 65% | 156 | 2.6E-10 |
| **Translation, ribosomal structure and biogenesis** | | | |  |  |  |  |  |  |  |
|  | H96 | ORF0579 | ZP_06380226 | Putative transcriptional regulator [*Arthrospira* str. Paraca] | 20474/23839 | 4.91/5.42 | 11 | 73% | 111 | 8.3e-06 |
|  |  |  |  |  |  |  |  |  |  |  |
| Spot(0.02M) | Spot(1.0M) | ORF | Accession NO. | Gene Product (best hit in ncbinr database) | Theoretical/Experimental | | Matched Peptides | Cov | MASCOT Score | E-value |
|  |  |  |  |  | MW（kDa） | pI |  |  |  |  |
|  | H372 | ORF4554 | ZP_06381260 | Phage shock protein A, pspa [*Arthrospira* str. Paraca] | 28183/37833 | 5.02/4.43 | 12 | 53% | 74 | 0.042 |
| N210 |  | ORF5117 | ZP_06381795 | Ribosome recycling factor [*Arthrospira* str. Paraca] | 20263/26103 | 5.71/6.12 | 10 | 59% | 140 | 1.00E-08 |
| N283 |  | ORF4662 | ZP_03271593 | Sigma 54 modulation protein/ribosomal protein S30EA [*Arthrospira maxima* CS-328] | 23973/29221 | 7.03/6.22 | 9 | 58% | 94 | 0.00044 |
| N377 |  | ORF1074 | ZP_06380559 | Elongation factor TS [*Arthrospira* str. Paraca] | 24626/32194 | 5.74/6.12 | 7 | 53% | 75 | 0.034 |
| **Function unknown** | |  |  |  |  |  |  |  |  |  |
|  | H69 | ORF4634 | ZP_06380686 | Phycocyanin, alpha subunit [*Arthrospira maxima* CS-328] | 17703/19259 | 5.82/5.89 | 10 | 58% | 91 | 0.00076 |
|  | H352 |  |  |  |  |  |  |  |  |  |
| N18 |  |  |  | Hypothetical protein Rmar_0102 [*Rhodothermus marinus* DSM 4252] | 25840/28842 | 7.88/6.13 | 7 | 0.31 | 74 | 0.04 |
| N61 |  |  |  |  |  |  |  |  |  |  |
| N78 |  | ORF4030 | ZP_03276569 | Hypothetical protein aplap_24737 [*Arthrospira* str. Paraca] | 17324/16312 | 5.96/6.14 | 19 | 86% | 254 | 4.10E-20 |
| N89 |  |  |  | Hypothetical protein aplap_12998 [*Arthrospira* str. Paraca] | 19302/16685 | 9.47/4.10 | 14 | 72% | 187 | 2.10E-13 |
| N98 |  | ORF4030 | ZP_03276569 | Hypothetical protein aplap_24737 [*Arthrospira* str. Paraca] | 17324/17190 | 5.96/6.50 | 18 | 82% | 214 | 4.10E-16 |
| N184 |  |  |  |  |  |  |  |  |  |  |
| N266 |  |  |  |  |  |  |  |  |  |  |
| Spot(0.02M) | Spot(1.0M) | ORF | Accession NO. | Gene Product (best hit in ncbinr database) | Theoretical/Experimental | | Matched Peptides | Cov | MASCOT Score | E-value |
|  |  |  |  |  | MW（kDa） | pI |  |  |  |  |
| N339 |  | ORF0361 | ZP_06384062 | Peptidase S8 and S53 subtilisin kexin sedolisin [*Arthrospira* str. Paraca] | 44238/31219 | 4.59/4.65 | 16 | 44% | 200 | 1.00E-14 |
| N385 |  | ORF2155 | ZP_06382427 | Phycobilisome linker polypeptide [*Arthrospira* str. Paraca] | 29450/32400 | 9.25/5.26 | 23 | 67% | 269 | 1.3E-21 |
| N410 |  | ORF2155 | ZP_06382427 | Phycobilisome linker polypeptide [*Arthrospira* str. Paraca] | 29450/33455 | 9.25/6.40 | 15 | 54% | 186 | 2.60E-13 |
| N423 |  | ORF4633 | ABV01983 | Cpch [*Arthrospira* Sp-16] | 30852/33929 | 7.82/6.29 | 25 | 60% | 295 | 3.30E-24 |
| N424 |  | ORF5516 | ZP_03272395 | Conserved hypothetical protein [*Arthrospira maxima* CS-328] | 41116/34016 | 6.04/6.08 | 13 | 50% | 205 | 3.30E-15 |
| N784 |  | ORF4030 | AAS78467 | Hypothetical protein aplap_24737 [*Arthrospira* str. Paraca] | 17324/49227 | 5.96/6.07 | 19 | 86% | 254 | 4.10E-20 |

**Supplementary Table 4-E**

The MS analytical results of differential protein spots (>3-fold) between 1.0 M and 0.5 M NaCl treatment

| Spot(0.5M) | Spot(1.0M) | ORF | Accession NO. | Gene Product (best hit in ncbinr database) | Theoretical/Experimental | | Matched Peptides | Cov | MASCOT Score | E-value |
| --- | --- | --- | --- | --- | --- | --- | --- | --- | --- | --- |
|  |  |  |  |  | MW（kDa） | pI |  |  |  |  |
| **Coenzyme metabolism** | | |  |  |  |  |  |  |  |  |
| M747 | H518 | ORF4544 | ZP_06383181 | Coproporphyrinogen III oxidase [*Arthrospira* str. Paraca] | 39917/43886 | 5.17/5.01 | 14 | 40% | 106 | 2.60E-05 |
| M778 | H545 | ORF1688 | ZP_06381331 | S-adenosyl-L-homocysteine hydrolase [*Arthrospira* str. Paraca] | 46667/45550 | 5.64/5.98 | 25 | 52% | 245 | 3.3e-19 |
| **Posttranslational modification, protein turnover, chaperones** | | | | |  |  |  |  |  |  |
| M908 | H653 | ORF2287 | ZP_03274434 | Chaperone protein dnak [*Arthrospira maxima* CS-328] | 62452/57079 | 4.70/5.57 | 27 | 38% | 150 | 1.00E-09 |
| **Inorganic ion transport and metabolism** | | | |  |  |  |  |  |  |  |
| M800 | H564 | ORF2050 | ZP_03275033 | ABC-type nitrate/nitrite transport system substrate-binding protein [*Arthrospira maxima* CS-328] | 48859/47107 | 4.68/4.46 | 26 | 50% | 235 | 3.30E-18 |
| **Secondary metabolites biosynthesis, transport, and catabolism / General function prediction only** | | | | | | | |  |  |  |
| M344 | H224 | ORF1433 | YP_002521548 | Oxidoreductase, short-chain dehydrogenase/reductase family [*Thermomicrobium roseum* DSM 5159] | 28631/31269 | 6.54/5.34 | 9 | 34% | 73 | 0.049 |
| **RNA processing and modification** | | | |  |  |  |  |  |  |  |
| M839 | H591 | ORF0624 | ZP_06384828 | Hypothetical protein aplap_24525 [*Arthrospira* str. Paraca] | 37661/50381 | 4.91/5.25 | 27 | 61% | 226 | 2.60E-17 |
| Spot(0.5M) | Spot(1.0M) | ORF | Accession NO. | Gene Product (best hit in ncbinr database) | Theoretical/Experimental | | Matched Peptides | Cov | MASCOT Score | E-value |
|  |  |  |  |  | MW（kDa） | pI |  |  |  |  |
| **Transcription / Signal transduction mechanisms** | | | |  |  |  |  |  |  |  |
| M542 | H364 | ORF4554 | ZP_06381260 | Phage shock protein A, pspa [*Arthrospira* str. Paraca] | 28183/38170 | 5.02/4.64 | 16 | 50% | 97 | 0.0002 |
| **Function unknown** | |  |  |  |  |  |  |  |  |  |
| M565 | H380 | ORF0623 | ZP_06380200 | Hypothetical protein aplap_00795 [*Arthrospira* str. Paraca] | 38307/38980 | 5.03/5.04 | 27 | 65% | 146 | 2.60E-09 |

**Supplementary Table 4-F**

The MS analytical results of the sole present protein spots in 1.0 M NaCl or 0.5 M treatment

| Spot(0.5M) | Spot(1.0M) | ORF | Accession NO. | Gene Product (best hit in ncbinr database) | Theoretical/Experimental | | | Matched Peptides | Cov | MASCOT Score | E-value |
| --- | --- | --- | --- | --- | --- | --- | --- | --- | --- | --- | --- |
|  |  |  |  |  | MW（kDa） | | pI |  |  |  |  |
| **Acyl-carrier-protein** | |  |  |  |  | |  |  |  |  |  |
| M361 |  | ORF5055 | ZP_06383266 | Enoyl-(acyl carrier protein) reductase [*Arthrospira* str. Paraca] | 27751/31814 | | 5.57/5.83 | 11 | 37% | 79 | 0.013 |
| **Amino acid transport and metabolism / Cell envelope biogenesis, outer membrane** | | | | | | | |  |  |  |  |
| M384 |  | ORF0089 | ZP_06380542 | Diaminopimelate epimerase [*Arthrospira* str. Paraca] | 31569/32349 | | 4.97/4.95 | 21 | 71% | 177 | 2.10E-12 |
| **Cell envelope biogenesis, outer membrane** | | | |  |  | |  |  |  |  |  |
| M290 |  | ORF4030 | AAS78467 | Gvpc [*Microcystis* sp. FACHB-854] | 21040/29650 | | 9.45/6.64 | 6 | 36% | 76 | 0.025 |
| **Cell envelope biogenesis, outer membrane / Carbohydrate transport and metabolism** | | | | | | | |  |  |  |  |
| M134 |  | ORF4043 | ZP_03274588 | dTDP-4-dehydrorhamnose 3,5-epimerase [*Arthrospira maxima* CS-328] | 20785/22132 | | 5.00/5.24 | 13 | 70% | 138 | 1.7E-08 |
| **Cell division and chromosome partitioning** | | | |  |  | |  |  |  |  |  |
| M125 |  | ORF0639 | ZP_02928939 | FHA domain containing protein [*Verrucomicrobium spinosum* DSM 4136] | 170844/21007 | | 5.09/5.86 | 27 | 19% | 84 | 0.0041 |
| **Cell motility and secretion** | | |  |  |  | |  |  |  |  |  |
|  | H185 | ORF4213 | YP_798213 | Flagellar biosynthesis regulator flhf [*Leptospira borgpetersenii* serovar Hardjo-bovis L550] | 49178/28565 | | 7.08/5.46 | 8 | 24% | 74 | 0.046 |
| Spot(0.5M) | Spot(1.0M) | ORF | Accession NO. | Gene Product (best hit in ncbinr database) | Theoretical/Experimental | | | Matched Peptides | Cov | MASCOT Score | E-value |
|  |  |  |  |  | MW（kDa） | pI | |  |  |  |  |
| **Energy production and conversion** | | | |  |  | |  |  |  |  |  |
| M371 |  | ORF2754 | ZP_06385149 | NADH dehydrogenase subunit B [*Arthrospira* str. Paraca] | 27251/32011 | | 6.75/6.69 | 16 | 48% | 191 | 8.3e-14 |
| **General function prediction only** | | | |  |  | |  |  |  |  |  |
|  | H138 | ORF1601 | ZP_02733328 | NB-ARC domain protein [*Gemmata obscuriglobus* UQM 2246] | 53287/26302 | | 8.98/4.66 | 13 | 24% | 82 | 0.0065 |
| M313 |  | ORF3836 | ZP_06383853 | Exsb protein [*Arthrospira* str. Paraca] | 24980/30364 | | 5.21/5.33 | 13 | 60% | 98 | 0.00015 |
| **Posttranslational modification, protein turnover, chaperones** | | | | |  | |  |  |  |  |  |
|  | H91 | ORF1368 | ZP_03273475 | Redoxin domain protein [*Arthrospira maxima* CS-328] | 19780/23134 | | 4.88/5.12 | 11 | 59% | 80 | 0.011 |
| M42 |  |  | ZP_02930161 | Protein-P-II uridylyltransferase, putative [*Verrucomicrobium spinosum* DSM 4136] | 107860/15792 | | 8.67/4.36 | 22 | 26% | 75 | 0.033 |
| M113 |  | ORF1746 | ZP_06383547 | Alkyl hydroperoxide reductase/ Thiol specific antioxidant/ Mal allergen [*Arthrospira* str. Paraca] | 16988/19692 | | 5.07/4.99 | 8 | 71% | 108 | 1.7e-05 |
| M369 |  | ORF1091 | ZP_06380727 | Methionine sulfoxide reductase A [*Arthrospira* str. Paraca] | 26421/31893 | | 6.52/6.27 | 11 | 26% | 93 | 0.00051 |
| **Inorganic ion transport and metabolism** | | | |  |  | |  |  |  |  |  |
| M137 |  | ORF1911 | ZP_06383116 | Adenylylsulfate kinase [*Arthrospira* str. Paraca] | 19897/22320 | | 5.22/5.46 | 16 | 79% | 200 | 1e-14 |
| **Nucleotide transport and metabolism** | | | |  |  | |  |  |  |  |  |
|  |  |  |  |  |  | |  |  |  |  |  |
| Spot(0.5M) | Spot(1.0M) | ORF | Accession NO. | Gene Product (best hit in ncbinr database) | Theoretical/Experimental | | | Matched Peptides | Cov | MASCOT Score | E-value |
|  |  |  |  |  | MW（kDa） | | pI |  |  |  |  |
| M8 |  | ORF1392 | YP_589310 | Phosphoribosylamine--glycine ligase [*Candidatus Koribacter versatilis* Ellin345] | 45762/41232 | | 7.13/6.56 | 14 | 36% | 73 | 0.05 |
| **Secondary metabolites biosynthesis, transport, and catabolism / General function prediction only** | | | | | | | | |  |  |  |
| M302 |  | ORF2597 | ZP_06383388 | Short-chain dehydrogenase/reductase SDR [*Arthrospira* str. Paraca] | 19509/30155 | | 5.54/6.48 | 17 | 73% | 185 | 3.3e-13 |
| **Transcription / Signal transduction mechanisms** | | | |  |  | |  |  |  |  |  |
|  | H372 | ORF4554 | ZP_06381260 | Phage shock protein A, pspa [*Arthrospira* str. Paraca] | 28183/37833 | | 5.02/4.43 | 12 | 53% | 74 | 0.042 |
| **Translation, ribosomal structure and biogenesis** | | | |  |  | |  |  |  |  |  |
| M184 |  | ORF5117 | ZP_06381795 | Ribosome recycling factor [*Arthrospira* str. Paraca] | 20263/25844 | | 5.71/6.02 | 18 | 77% | 161 | 8.3e-11 |
| **Transcriptional regulator** | | | | | | | | | | |  |
| M111 |  | ORF1989 |  | Transcriptional regulator, abrb family [*Arthrospira maxima* CS-328] | 15532/19479 | | 8.49/4.22 | 11 | 44% | 90 | 0.0011 |
| **Function unknown** | |  |  |  |  | |  |  |  |  |  |
|  | H69 | ORF4634 |  | Phycocyanin, alpha subunit [*Arthrospira maxima* CS-328] | 17703/19259 | | 5.82/5.89 | 10 | 58% | 91 | 0.00076 |
|  | H441 |  |  |  |  | |  |  |  |  |  |
| M78 |  | ORF4634 |  | Phycocyanin, alpha subunit [*Arthrospira maxima* CS-328] | 17703/17228 | | 5.82/5.99 | 11 | 74% | 78 | 0.015 |
| M109 |  | ORF5159 |  | Phycobilisome protein [*Arthrospira maxima* CS-328] | 17438 /19403 | | 4.89/4.52 | 14 | 61% | 113 | 5.2E-06 |
| M178 |  | ORF1456 | ZP_06380822 | Pentapeptide repeat-containing protein [*Arthrospira* str. Paraca] | 19860/25627 | | 5.13/5.42 | 12 | 47% | 77 | 0.021 |

**Supplementary Table 5**

**The standard curve equation, regression coefficient and amplified efficiency of the 16S rRNA genes**

| Name | Standard curve equation | Regression coefficient (R^2^) | Amplified efficiency% |
| --- | --- | --- | --- |
| 16S rRNA^1^ | y = -3.169x + 45.743 | 0.984 | 106.808 |
| 16S rRNA^2^ | y = -3.518x + 48.417 | 0.982 | 92.421 |
| 16S rRNA^3^ | y = -3.162x + 41.824 | 0.998 | 107.141 |
| 16S rRNA^4^ | y = -3.442x + 46.572 | 0.995 | 95.226 |
| 16S rRNA^5^ | y = -3.391x + 45.722 | 0.996 | 97.196 |
| 16S rRNA^6^ | y = -3.826x + 51.764 | 0.996 | 82.536 |
| 16S rRNA^7^ | y = -3.878x + 50.014 | 0.994 | 81.075 |
| 16S rRNA^8^ | y = -2.968x + 41.617 | 0.982 | 117.211 |
| 16S rRNA^9^ | y = -3.606x + 47.909 | 0.998 | 89.365 |
| 16S rRNA^10^ | y = -3.717x + 45.383 | 0.989 | 85.792 |
| 16S rRNA^11^ | y = -3.28x + 43.615 | 0.999 | 101.793 |
| 16S rRNA^12^ | y = -3.18x + 42.666 | 0.998 | 106.279 |
| 16S rRNA^13^ | y = -3.812x + 48.457 | 0.995 | 82.949 |
| 16S rRNA^14^ | y = -3652x +51.038 | 0.993 | 87.839 |

**Supplementary Table 6**

**The standard curve, regression coefficient and amplified efficiency of the target genes**

| ORF | Standard curve | Regression coefficient (R^2^) | Amplified efficiency% |
| --- | --- | --- | --- |
| 3277 | y = -3.122x + 46.061 | 0.981 | 109.053 |
| 2281 | y = -3.27x + 40.024 | 0.997 | 102.205 |
| 4535 | y = -3.483x + 54.438 | 0.98 | 93.691 |
| 5055 | y = -3.177x + 50.76 | 0.988 | 106.439 |
| 4103 | y = -3.405x + 48.26 | 0.997 | 96.638 |
| 1911 | y = -2.966x + 45.86 | 0.994 | 117.359 |
| 4633 | y = -3.675x + 45.622 | 0.992 | 87.125 |
| 2155 | y = -3.785x + 53.817 | 0.993 | 83.736 |
| 4030 | y = -3.169x + 44.537 | 0.98 | 106.798 |
| 1989 | y = -3.575x + 51.476 | 0.999 | 90.417 |
| 1251 | y = -3.299x + 48.484 | 0.983 | 100.957 |
| 5516 | y = -3.456x + 50.022 | 0.987 | 94.692 |
| 3023 | y = -3.761x + 51.918 | 0.988 | 84.454 |
| 3910 | y = -3.898x + 53.21 | 0.988 | 80.541 |
| 4938 | y = -3.557x + 50.924 | 0.992 | 91.049 |
| 3807 | y = -3.871x + 52.018 | 0.987 | 81.272 |
| 1688 | y = -3.652x + 51.038 | 0.993 | 87.839 |
| 2740 | y = -3.046x + 39.651 | 0.98 | 112.982 |
| 2739 | y = -3.291x + 50.224 | 0.988 | 101.32 |
| 1433 | y = -3.44x + 47.638 | 0.995 | 95.283 |
| 2287 | y = -3.486x + 45.796 | 0.982 | 93.581 |
| 2050 | y = -3.576x + 53.106 | 0.994 | 90.389 |
| 4213 | y = -3.894x + 53.931 | 0.98 | 80.625 |
| 4632 | y = -3.296x + 44.602 | 0.998 | 101.102 |
| 4634 | y = -3.622x + 45.964 | 0.996 | 88.832 |
| 4635 | y = -3.789x + 49.826 | 0.992 | 83.609 |
| 273 | y = -3.354x + 45.172 | 0.994 | 98.691 |
| 1198 | y = -3.571x + 50.722 | 0.99 | 90.554 |
| 624 | y = -3.219x + 47.544 | 0.992 | 104.473 |





**Supplementary Figure 1**

**ASP growth curve in different salt concentrations**


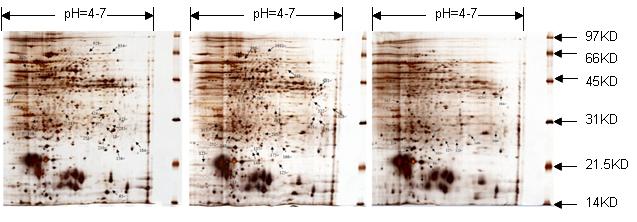


A-0.02M B-0.5M C-1.0M


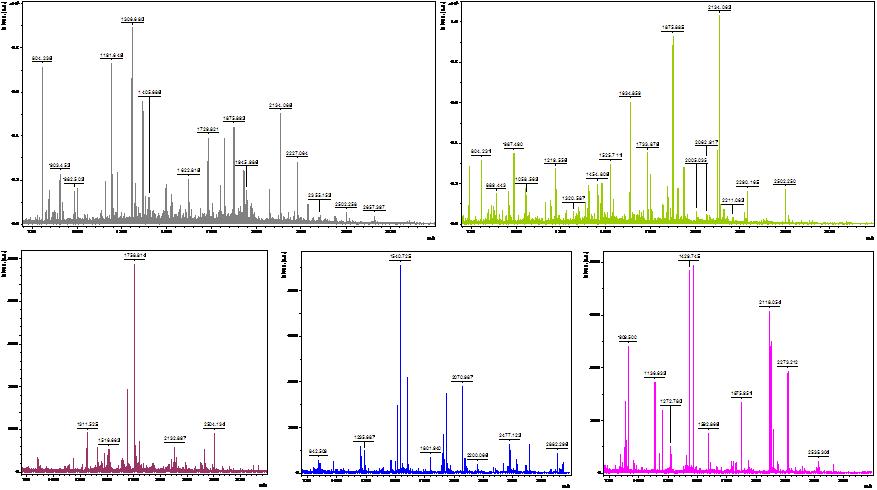


**Supplementary Figure 2**

**The differential protein profiles of 2-DE of ASP in control (0.02M), medium salt treatment**

**(0.5M) and high salt treatment (1.0M) groups**


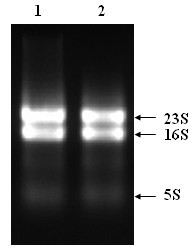


**Supplementary Figure 3**

**The AGE profile of total RNA in control and medium salt treatment.** (Note: Lane 1, control; Lane 2, medium salt treatment.)


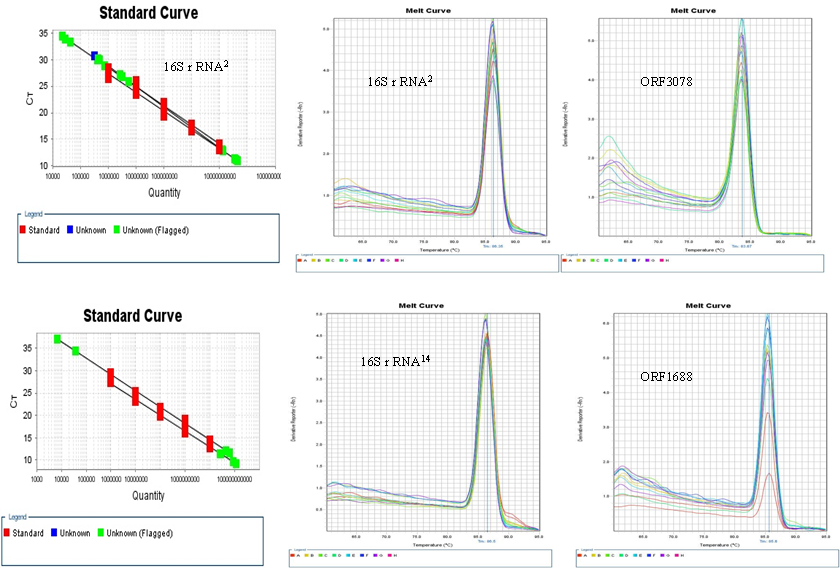


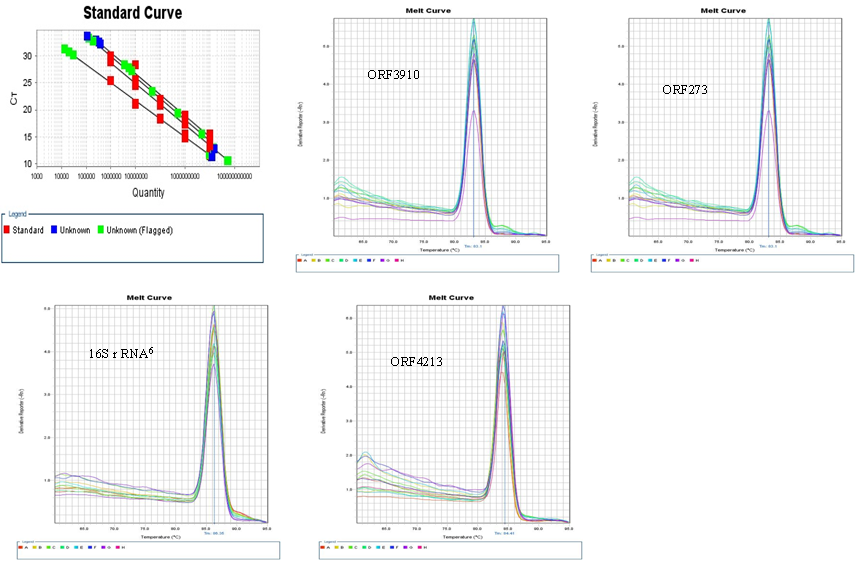


**Supplementary Figure 4**

**The standard and melting curve of 16S rRNA and sample**
